# A comparative analysis of mitochondrial ORFs provides new insights on expansion of mitochondrial genome size in Arcidae

**DOI:** 10.1186/s12864-022-09040-3

**Published:** 2022-12-07

**Authors:** Ning Zhang, Yuanning Li, Kenneth M. Halanych, Lingfeng Kong, Qi Li

**Affiliations:** 1 Key Laboratory of Mariculture, Ministry of Education, Ocean University of China, Qingdao, China; 2 Shandong University, Qingdao, China; 3 Center for Marine Science, University of North Carolina Wilmington, Wilmington, NC 28409 USA; 4 Laboratory for Marine Fisheries Science and Food Production Processes, Qingdao National Laboratory for Marine Science and Technology, Qingdao, China

**Keywords:** Arcidae, Mitochondrial genome size, Mitochondrial ORFs, Unassigned regions

## Abstract

**Background:**

Arcidae, comprising about 260 species of ark shells, is an ecologically and economically important lineage of bivalve mollusks. Interestingly, mitochondrial genomes of several Arcidae species are 2–3 times larger than those of most bilaterians, and are among the largest bilaterian mitochondrial genomes reported to date. The large mitochondrial genome size is mainly due to expansion of unassigned regions (regions that are functionally unassigned). Previous work on unassigned regions of Arcidae mtDNA genomes has focused on nucleotide-level analyses to observe sequence characteristics, however the origin of expansion remains unclear.

**Results:**

We assembled six new mitogenomes and sequenced six transcriptomes of *Scapharca broughtonii* to identify conserved functional ORFs that are transcribed in unassigned regions. Sixteen lineage-specific ORFs with different copy numbers were identified from seven Arcidae species, and 11 of 16 ORFs were expressed and likely biologically active. Unassigned regions of 32 Arcidae mitogenomes were compared to verify the presence of these novel mitochondrial ORFs and their distribution. Strikingly, multiple structural analyses and functional prediction suggested that these additional mtDNA-encoded proteins have potential functional significance. In addition, our results also revealed that the ORFs have a strong connection to the expansion of Arcidae mitochondrial genomes and their large-scale duplication play an important role in multiple expansion events. We discussed the possible origin of ORFs and hypothesized that these ORFs may originate from duplication of mitochondrial genes.

**Conclusions:**

The presence of lineage-specific mitochondrial ORFs with transcriptional activity and potential functional significance supports novel features for Arcidae mitochondrial genomes. Given our observation and analyses, these ORFs may be products of mitochondrial gene duplication. These findings shed light on the origin and function of novel mitochondrial genes in bivalves and provide new insights into evolution of mitochondrial genome size in metazoans.

**Supplementary Information:**

The online version contains supplementary material available at 10.1186/s12864-022-09040-3.

## Background

Mitochondria, specialized organelles of eukaryotic cells that possess their own genome (mitochondrial genome or mitogenome), have been traditionally described as cellular “power plants” for producing ATP [1]. Mitochondria were derived from an ancestral endosymbiotic alphaproteobacterium integrated into a host cell related to Asgard Archaea [2]. Subsequently the mitogenome experienced massive genome reductive evolution (GRE) [2, 3]. After that, mitochondria coevolved with different hosts and underwent both neutral modifications and adaptive responses that led to the diversity observed today in mitogenomes [4]. In bilaterians, mitogenomes were considered to be extremely compact and normally organized into a single circular molecule ranging in size from 14 to 20 kb [5]. Bilaterian mitogenomes typically contain the same set of 37 genes (13 protein-coding genes encoding different subunits of enzyme complexes for the oxidative phosphorylation (OXPHOS) system, 2 ribosomal RNAs (rrnS and rrnL), 22 transfer RNA genes) and no introns [5–7]. In the last few years, high-throughput sequencing techniques and extensive sampling for phylogenetic and population genetic studies have accelerated the sequencing of mitogenomes and uncovered the great diversity of structural features [8]. An increasing number of mitogenomes seem to deviate dramatically from typical bilaterian mitogenomes and present wide variation in genome size, and many of them are much larger than 20 kb. Many molluscs, especially bivalves, display an unusual amount of variation in mitogenome structure and size, even among closely related species [6, 9, 10].

Arcidae, known as Ark shell or blood cockles, are an economically-important group of bivalves and have a long evolutionary history, dating back to the Lower Ordovician ~ 450 Mya [11]. Interestingly, mitogenomes of Arcidae species both within and between species reveal a high variability in size, ranging from 19 to 56 kb in length [12]. For example, reported mitogenomes of *Scapharca broughtonii* are 46,985 bp [13] and 48,161 bp [14], the recently published *Scapharca gubernaculum* mitogenome is 45,697 bp [15], which are 2–3 times larger than other bilaterians. The largest Arcidae mitogenome comes from *Scapharca kagoshimensis* (46.7–56.2 kb) and is the largest bilaterian mitogenome yet recorded, out of approximately 86,900 mt-DNAs from more than 11,600 species [8, 12]. In addition, large mitogenomes are also found in sea scallop *Placopecten magellanicus* (31-41 kb) [16] and the clavagellid mussel *Bryopa lata* (32 kb) [17]. The large genome sizes in ark shells and sea scallop are not primarily a result of duplications of control region sequences and coding sequences like model organisms, but rather the expansion of unassigned regions (i.e., noncoding regions that are functionally unassigned) [16, 18]. A previous analysis [19] of 2656 complete mitogenomes showed that some bivalves have a proportion of unassigned regions (URs) that are significantly different from all other groups and show the highest median percentage of URs in Metazoans. According to the mutation pressure theory [20], fast evolving organelle genomes experience more selection pressure for genome reduction, but some bivalve mitogenomes seem to contradict this theory. In some Arcidae species, URs account for more than 50% of the entire mitogenomes. Tandem repeats, inverted repeats and transposable elements in unassigned regions (URs) have been shown to contribute to the large size of these mitogenomes [12, 13, 16], but it does not mean they are the main cause of huge expansion in URs. Data from previous studies show that repeat families and transposable elements are not the main components of large URs, which only account for 6–31% of URs in different Arcidae species, though they have a significantly positive correlation with mitogenome size [12, 13]. This suggests that there are other components influencing the size of URs. One possible explanation is that retention of the URs in bivalve mitogenomes is caused by the presence of functional sequences and/or structures. However, to date, much of the work on URs of Arcidae has focused on nucleotide-level analyses to observe sequence characteristics (e.g., tandem repeats, inverted repeats), mitogenome expansion remains poorly understood and needs further study with different perspectives.

Mitochondria are long known for bioenergetics, but they also have novel non-OXPHOS-related adaptations and functions [21]. With the increasing number of published mitogenomes, non-standard gene contents have been found in different animal groups, and additional mitochondrial protein-coding genes have been identified and annotated in mitogenomes of metazoan, particularly in invertebrates. For example, additional mitochondrial protein-coding genes were first discovered in the octocoral *Sarcophyton glaucum* [22], which was a homolog of *mutS* and hypothesized to originate either through bacterium or viral infection by horizontal gene transfer [23, 24]. In cnidarians, sponges and placozoans, protein-coding genes with non-OXPHOS functions (e.g., *dnaB*, *tatC*) have been also reported [25, 26]. Surprisingly, nine additional mtDNA-encoded protein genes have been described in humans [27–30], one of which is a 75 bp ORF in the mitochondrial 16S rRNA that acts as a neuroprotector, an antiapoptotic agent, and a cytoprotector [31, 32]. These discoveries indicate that there are additional functional sequences in mitochondria, maybe related to its diverse functions.

Moreover, multiple ORF sequences have also been found in the mitogenomes of bivalves. A novel ORF was discovered with no sequence- or domain-based homology to mitochondrial genes in the mitogenome of pearl-lip oyster *Pinctada maxima* but has domain-based homology to the nuclear genome [33]. Mitochondrial ORFans (open reading frames having no detectable homology and no known function) also have been identified in marine and freshwater bivalves (Mytiloida, Nuculanoida, Unionoida, and Veneroida) with doubly uniparental inheritance (DUI) of mitochondrial DNA [34, 35]. In these cases, products are exported from the organelle and may be involved in functions other than energy production [34–40]. These studies indicate that traditional bivalve mitochondrial non-coding regions have sequences or unassigned regions that potentially perform biological functions. The structure of some special ORFs in the mitogenome of *Tegillarca granosa* (Arcidae) have been briefly investigated [41], but the origin of Arcidae ORFs in large URs remain unclear. In addition, a fundamental question regarding the size of mitogenomes in Arcidae bivalves is whether there are ORFs in large URs that perform functions and have a connection with expanded size of mitogenomes.

Here, we sequenced and annotated five new mitogenomes (four *S. broughtonii* and one *S. cornea*) and assembled a mitogenome of *S. kagoshimensis* from NCBI data (SRX8857271). Six transcriptomes of *S. broughtonii* were sequenced and analyzed to identify conserved functional ORFs. Multiple samples from the same species were used to detect intraspecific variation in mitogenome length and the presence of ORFs. To better understand how URs expand and evolved in Arcidae, we present a comparative analysis of 32 complete mitogenomes (6 new assemblies and 26 published assemblies from NCBI) of Arcidae species to highlight both unique features and characteristics shared among different species, with an emphasis on characterizing large URs and ORFs. Then, we investigated the origin and duplication of ORFs and their correlation with mitogenome expansion, and particularly with the expansion and function of mitochondrial large URs in Arcidae.

## Results

### Mitogenome assembly, annotation and features

**Fig. 1:**
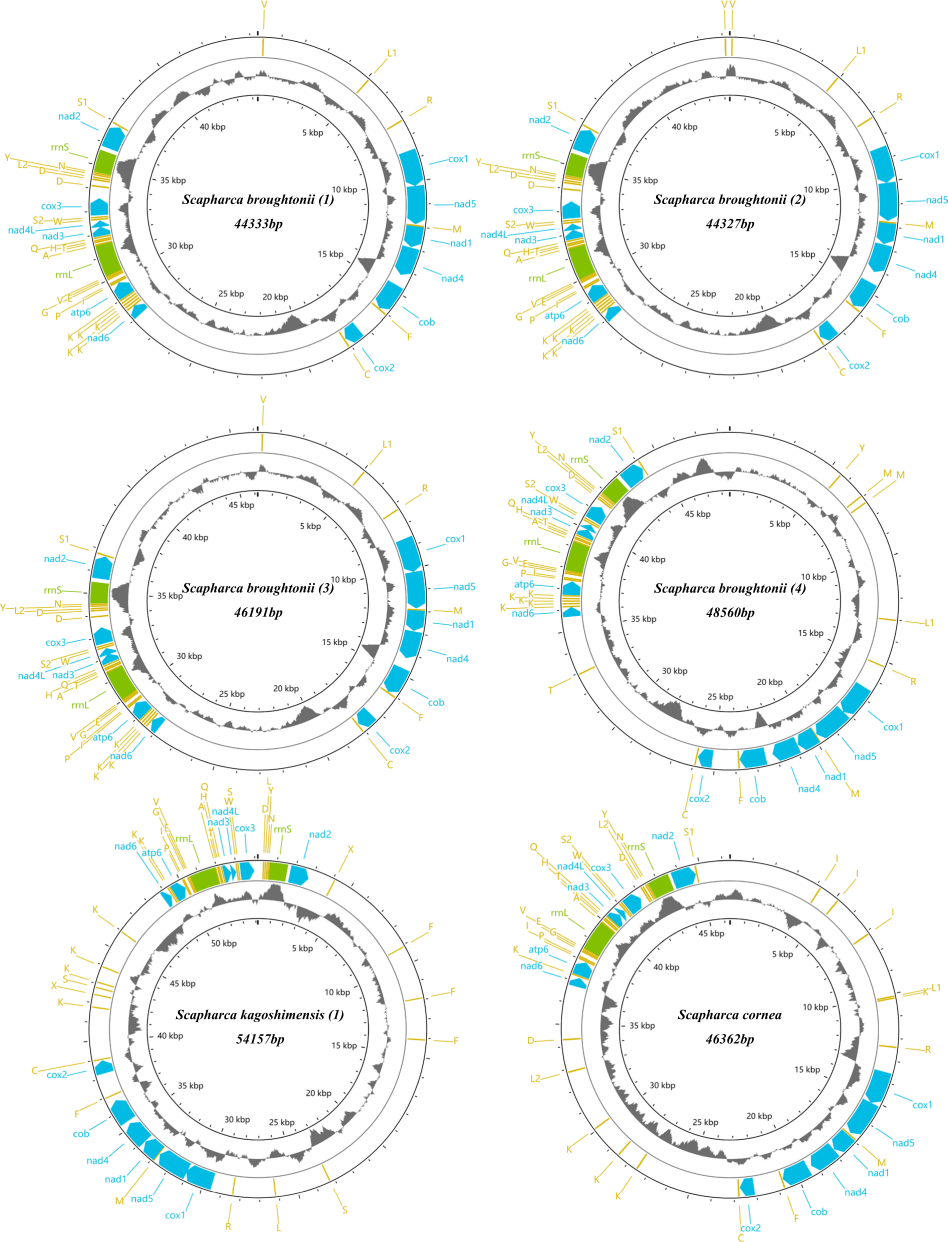
Maps of the six mitogenomes sequenced in this study. The corresponding species name and length are given inside each genome map. The outer ring comprises all standard and putative coding sequences, identified with the following color code: blue, genes encoding electron transport chain and ATP-synthase subunits; yellow, tRNA genes; green, rRNA genes (see Additional file 1: Table S1 for details). All the genes are encoded on the same strand. The middle ring represents GC content (dark grey). The inner ring represents scale

**Fig. 2:**
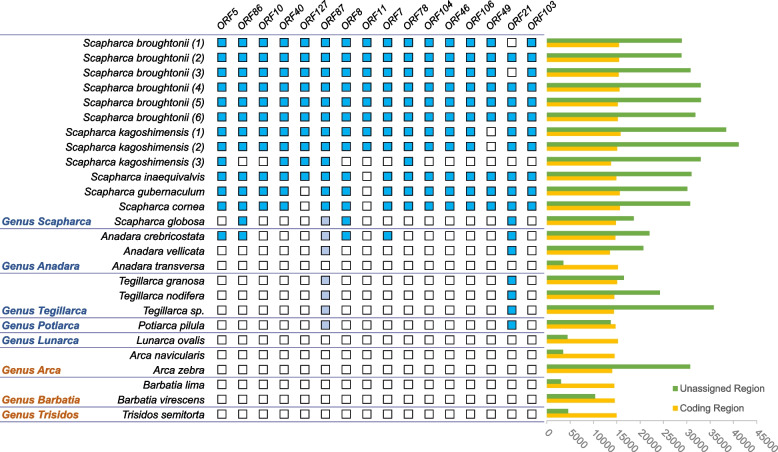
Distribution of ORF genes identified (middle) and size of coding region and unassigned region (right). Presence and absence of corresponding ORFs in Arcidae mitogenomes is shown by presence and absence of boxes, respectively. Blue boxes indicate ORFs completely annotated in mitogenomes. Light purple boxes indicate ORFs partially aligned in mitogenomes. Yellow bars indicate the length of coding region. Green bars indicate the length of unassigned region

Complete mitogenomes sequences of four *S. broughtonii*, one *S. kagoshimensis* and one *S. cornea* had sequence lengths more than 40 kb (Table 1). Four new *S. broughtonii* mitogenomes sequences varied in size from 44,327 bp to 48,560 bp, close to previously published *S. broughtonii* mitogenome (46,985 bp) [13]. The length of *S. kagoshimensis* mitogenome reported here was 54,157 bp, slightly smaller than the previously reported *S. kagoshimensis* (56,170 bp) [12], and *S. cornea* is 46,362 bp long. *S. broughtonii* and *S. kagoshimensis* mitogenomes vary dramatically in length within species. All mitogenomes consisted of 12 protein-coding genes (all taxa lacked *atp*8), two ribosomal RNA genes (*rrnS* and *rrnL*) and 27–33 tRNA genes (Additional file 1: Table S1). *Atp8* has never been found in Arcidae species [12, 18, 41, 42]. All protein-coding genes and rRNA genes in the six mitogenomes are encoded on the same strand and share the same gene order. In addition, a duplication of *cox2* was observed in all six mitogenomes (Additional file 1: Table S2), located between *cob* and *cox*2. The two copies have different length: the *cox*2 is 666–720 bp long (221-239aa), while the *cox*2-b is 1179–1431 bp long (392-476aa). This indicates that *cox*2-b have acquired an extension after *cox*2 duplication. Four different start codons (ATG, ATA, ATT, GTG) were observed but most protein-coding genes start with the codon ATG, and stop with the TAA and TAG codons. The organization of tRNAs was variable across the six mitochondrial genomes sequenced here. All mitogenomes are composed of four major segments: two coding regions and two major unassigned regions (Fig. 1). There is little variation in length of coding regions and great variation in URs (Fig. 2). These newly sequenced complete mitogenomes were deposited in GenBank (Accession numbers: OM807131-OM807136).

**Table 1:** Mitochondrial (mt) genomes analyzed in this study, including newly assembled mitogenomes and those from Genbank

Species	Subfamily	Length (bp)	SRA	Locality
New mt genomes				
*Scapharca broughtonii* (1)	Anadarinae	44,333		Qingdao, Shandong, China
*Scapharca broughtonii* (2)	Anadarinae	44,327		Qingdao, Shandong, China
*Scapharca broughtonii* (3)	Anadarinae	46,191		Qingdao, Shandong, China
*Scapharca broughtonii* (4)	Anadarinae	48,560		Qingdao, Shandong, China
*Scapharca cornea*	Anadarinae	46,362		Philippines
*Scapharca kagoshimensis* (1)	Anadarinae	54,157		Qingdao, Shandong, China
Species	Family/Subfamily	Length (bp)	GenBank acc. no.	Publication
GenBank mt genomes				
*Anadara crebricostata*	Anadarinae	36,671	MN316632	Kong et al., [12]
*Anadara transversa*	Anadarinae	18,780	MN326817	Kong et al., [12]
*Anadara vellicata*	Anadarinae	34,147	KP954700	Sun et al., [42]
*Lunarca ovalis*	Anadarinae	19,620	MN366010	Kong et al., [12]
*Potiarca pilula*	Anadarinae	28,386	KU975162	Sun et al., [43]
*Scapharca broughtonii* (5)	Anadarinae	48,161	KF667521	Hou et al., [14]
*Scapharca broughtonii* (6)	Anadarinae	46,985	AB729113	Liu et al., [13]
*Scapharca globosa*	Anadarinae	33,405	MN366011	Kong et al., [12]
*Scapharca gubernaculum*	Anadarinae	45,697	MN061840	Sun et al., [15]
*Scapharca inaequivalvis*	Anadarinae	45,859	MN366012	Kong et al., [12]
*Scapharca kagoshimensis* (2)	Anadarinae	56,170	MN366013	Kong et al., [12]
*Scapharca kagoshimensis* (3)	Anadarinae	46,713	KF750628	Sun et al., [18]
*Tegillarca* sp.	Anadarinae	50,104	MN366016	Kong et al., [12]
*Tegillarca granosa*	Anadarinae	31,589	KJ607173	Sun et al., [41]
*Tegillarca nodifera*	Anadarinae	38,672	MN366014	Kong et al., [12]
*Arca navicularis*	Arcinae	18,004	MN326818	Kong et al., [12]
*Arca zebra*	Arcinae	44,651	MN366003	Kong et al., [12]
*Barbatia lima*	Arcinae	17,479	MN366005	Kong et al., [12]
*Barbatia virescens*	Arcinae	24,871	MN366006	Kong et al., [12]
*Trisidos semitorta* (1)	Arcinae	19,461	MN366015	Kong et al., [12]
*Trisidos semitorta* (2)	Arcinae	19,613	KU975161	Sun et al., [43]
*Cucullaea labiate* (1)	Cucullaeididae	25,845	KP091889	Feng et al., [44]
*Cucullaea labiate* (2)	Cucullaeididae	20,481	MN366007	Kong et al., [12]
*Glycymeris formosana*	Glycymerididae	19,027	MN366008	Kong et al., [12]
*Glycymeris yessoensis*	Glycymerididae	17,903	MN366009	Kong et al., [12]
*Arcopsis adamsi*	Noetiidae	18,716	MN366004	Kong et al., [12]
Outgroup				
*Mizuhopecten yessoensis*	Pectinidae	20,964	FJ595959.1	Wu et al., [45]
*Pinctada maxima*	Pteriidae	16,994	NC_018752.1	Wu et al., [46]
*Crassostrea gigas*	Ostreidae	18,224	AF177226.1	N/A

### Characterization of unassigned regions in mitogenomes

All available mitogenomes of *S. broughtonii*, *S. kagoshimensis* and *S. cornea* were characterized by large unassigned regions separated into two principal blocks. The first block (UR1) was located between *cox*2 and *nad*6, and the second block (UR2) was located between *nad*2 and *cox*1. We refer to these as “shared large unassigned regions”. An assessment of shared URs in nine mitogenomes (Table 2) revealed that the length of UR1 is relatively stable between 9831 and 10,120 bp except in *S. cornea* where it was 14,034 bp. UR2 length was highly variable from 15,167 to 28,331 bp, and the intergenic DNA (URs between genes in coding blocks) ranged from 1507 to 2925 bp. Overall, the total length of URs was 28,844 to 41,101 bp, accounting for 65.1–73.2% of these nine mitogenomes. Tandem repeats in URs showed significant variation in number (2–26 copies) and sequence length (1-273 nt) (Additional file 1: Table S3). The total length of tandem repeats varied from 759 bp to 4942 bp and takes up only 2–15% of URs. In addition, an examination of all Arcidae complete mitogenomes showed that unassigned regions (i.e., repeats, ORFs) were highly variable and responsible for expansion and variation in Arcidae mitogenomes (Additional file 1: Table S4). In comparison, a higher proportion of unassigned sequences was observed in the ark shell of *Scapharca* (> 60%) and *Tegillarca* (> 50%), both exceeding 16 kbp, and thus there was a strong positive correlation between mitogenome size and proportion of unassigned region.

**Table 2:** Detailed information of unassigned regions of Arcidae mitogenomes. The unit of length is the bp

Species	Mitogenome size	The total length of URs	Proportion of URs	UR1 location	UR1 size	Proportion of UR1	UR2 location	UR2 size	Proportion of UR2
*Scapharca broughtonii* (1)	44,333	28,908	65.21%	18,057–27,994	9938	22.42%	1–8614 36,903–44,333	16,045	36.19%
*Scapharca broughtonii* (2)	44,327	28,844	65.07%	17,914–27,851	9938	22.42%	1–8471 36,760–44,327	16,039	36.18%
*Scapharca broughtonii* (3)	46,191	30,806	66.69%	18,033–27,968	9936	21.51%	1–8590 36,753–46,191	18,029	39.03%
*Scapharca broughtonii* (4)	48,560	32,984	67.92%	25,845–35,677	9833	20.25%	1–16,532 44,063–48,560	21,030	43.31%
*Scapharca broughtonii* (5)	48,161	33,004	68.53%	9314–19,144	9831	20.41%	27,798–48,161	20,364	42.28%
*Scapharca broughtonii* (6)	46,985	31,809	67.70%	9314–19,146	9833	20.93%	27,673–46,985	19,313	41.11%
*Scapharca kagoshimensis* (1)	54,157	38,394	70.89%	38,928–48,778	9851	25.66%	2805–29,551	26,747	69.67%
*Scapharca kagoshimensis* (2)	56,170	41,101	73.17%	30,713–40,833	10,120	18.02%	1–21,329 49,169–56,170	28,331	50.44%
*Scapharca kagoshimensis* (3)	46,713	32,982	70.61%	9395–19,345	9951	21.30%	28,023–46,713	18,691	40.01%
*Scapharca cornea*	46,362	30,708	66.24%	22,735–36,777	14,034	30.27%	1–13,571 44,767–46,362	15,167	32.71%
*Scapharca gubernaculum*	45,697	30,079	65.82%	9164–21,106	11,943	26.14%	29,095–45,697	16,603	36.33%
*Scapharca globosa*	33,405	18,631	55.77%	14,724–17,921	3198	9.57%	1–4372 26,555–33,405	11,223	33.60%
*Anadara crebricostata*	36,671	21,991	59.97%	23,496–27,840	4345	11.85%	1–5933 36,253–36,671	6352	17.32%
*Anadara vellicata*	34,147	20,659	60.50%	9130–9424	295	0.86%	18,084–34,147	16,064	47.04%

### Novel ORFs in mitochondrial unassigned regions

**Fig. 3:**
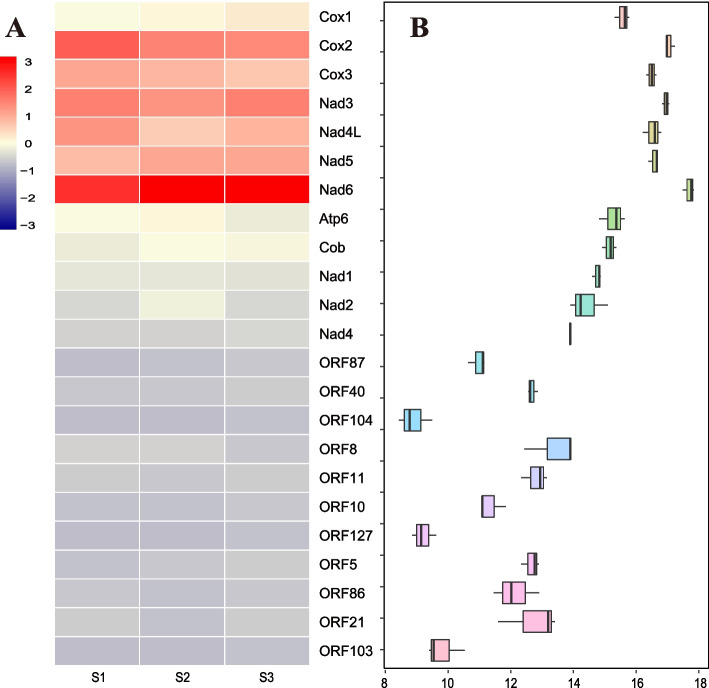
Transcription level of mitochondrial protein coding genes and ORFs. A: Heatmaps of genes and ORFs expressed between three different adductor muscle tissue of *S. broughtonii* from the same batch (S1, S2 and S3). B: Boxplots of transcription level of genes and ORFs. On the y axis is the name of genes and ORFs. On the x axis is plotted the log2 TPM value

TransDecoder (https://github.com/TransDecoder/TransDecoder/wiki) predicted eleven ORFs in mitochondrial unassigned regions that might code proteins from the *S. broughtonii* transcriptome. The analysis of mtDNA transcriptome expression (Fig. 3) showed that 12 mitochondrial coding genes have higher transcription level than all ORFs but ORF8 and ORF21 have a similar transcription level to *nad4*. *Nad6* had the highest transcription level of all PCGs and ORFs. In comparison, ORF104 and ORF127 showed a very low transcription level, which was considerably lower than other ORFs. The mapped read counts and TPM values of PCGs and ORFs have been recorded in Additional file 2. The ORFs shared the same location and order in four *S. broughtonii* mitogenomes (Additional file 1: Table S1). All were located in URs on the heavy strand (as all standard coding genes) of *S. broughtonii* mitogenomes, including six in UR1 and five in UR2. The eleven ORFs showed remarkable conservation in all samples: their start and stop codons were the same, respectively, except ORF127 in *S. broughtonii* (1), ORF21 in *S. broughtonii* (2) and ORF8 in *S. broughtonii* (3) (Table 3). The longest length of ORFs was 1983 bp (ORF87, 660aa), the shortest length of ORFs was 238 bp (ORF21, 79aa). Notably, the ORF8 was variable in its length (561 bp to 636 bp). Although *atp8* has not been previously reported in Arcidae, the lengths of ORF21 (238 bp) and ORF103 (244 bp) are close to that of *atp8* found in other bivalves (102–339 bp) and these ORFs may be candidates for *atp8*. Moreover, we found potential ORF duplication events in four *S. broughtonii* mitogenomes. Amino acid sequence comparisons using a combination of sequence and position similarity revealed that there are five additional ORFs that share a large degree of similarity within the eleven ORFs (Additional file 1: Table S5). Results showed that 47% amino acid identities were observed between ORF8 and ORF11, 39% for ORF8 and ORF7, 30% for ORF8 and ORF78. The highest identity (67% amino acid identity) was observed between ORF87 and ORF127. ORF104 had at least 28% amino acid identities with other three ORFs. These findings showed that ORFs have high similarity, suggesting that duplication events have occurred between them. However, no significant amino acid sequence similarity was detected with known proteins for the 14 new lineage-specific ORFs using BLAST [47] and PSI-BLAST against NRDB and UniProt (Additional file 1: Table S6).

**Table 3:** ORFs predicted by transcriptome and ORF Finder from four *Scapharca broughtonii* mitogenomes. Sb1, *Scapharca broughtonii* (1). The rest of the abbreviations are the same

ORF	Length (bp)				Initiation and termination codon			
	Sb1	Sb2	Sb3	Sb4	Sb1	Sb2	Sb3	Sb4
ORFs predicted by transcriptome								
ORF87	1983	1983	1983	1983	ATG-TAG	ATG-TAG	ATG-TAG	ATG-TAG
ORF40	819	831	831	831	ATG-TAG	ATG-TAG	ATG-TAG	ATG-TAG
ORF104	513	513	513	513	ATA-TAG	ATA-TAG	ATA-TAG	ATA-TAG
ORF8	609	636	561	636	ATA-TAA	ATA-TAA	ATG-TAA	ATA-TAA
ORF10	585	585	585	585	ATG-TAG	ATG-TAG	ATG-TAG	ATG-TAG
ORF11	603	603	603	603	ATA-TAA	ATA-TAG	ATA-TAG	ATA-TAG
ORF127	1809	1872	1872	1872	ATT-TAG	ATG-TAG	ATG-TAG	ATG-TAG
ORF5	762	762	762	762	ATA-TAA	ATA-TAA	ATA-TAA	ATA-TAA
ORF86	582	582	582	582	ATA-TAG	ATA-TAG	ATA-TAG	ATA-TAG
ORF21	–	238	–	238	–	ACG-T	–	ATG-T
ORF103	244	244	244	246	ATG-T	ATG-T	ATG-T	ATG-TAA

To establish whether these ORFs were taxonomically restricted to *S. broughtonii,* or if they were an evolutionary feature of ark shell, we screened for the presence of 16 ORFs (11 predicted ORFs and 5 duplicated ORFs, see Table 3 and Additional file 1: Table S5) in 32 mitogenomes of Arcidae (Table 1) with BLAST (Additional file 1: Table S7 and Additional file 3). None of the ORFs were similar to mitochondrial PCGs. Complete ORFs and their duplications could only be annotated in 13 mitogenomes, including 6 *Scapharca* and 1 *Anadara* species (Additional file 1: Table S8 and Fig. 2), suggesting that most of the ORFs were specific to the *Scapharca* lineage (except for *S. globosa*). With one exception, ORF21 was annotated in 12 species, which had a wider distribution. All 16 ORFs were verified in the mitogenomes of *S. broughtonii* and *S. kagoshimensis* except for *S. kagoshimensis* (3). Although the mitogenome of *S. kagoshimensis* (3) have a long URs, only five complete ORFs could be annotated, which may be due to poor assembly quality of the mitogenome. The result showed that 15 ORFs were found in the mitogenome of *S. inaequivalvis* except for ORF 11. In the mitogenomes of *S. gubernaculum* and *S. cornea*, 14 ORFs were found. Only ORF8, ORF21 and ORF86 were found in *S. globosa* mitogenome. Most of ORFs were unique to the genus *Scapharca* and *A. crebricostata*, but the partial sequence (about 120aa) of ORF87 was similar to fragments of other genera mitogenomes (Additional file 1: Table S7), which suggests that ORF87 may have had a wider distribution in Arcidae (Fig. 2). Sequence comparisons both within and between lineage-specific ORFs revealed high variability in length, but some ORF lengths were conservative such as ORF10 and ORF11 (Additional file 1: Table S8). The total length of all ORFs identified in *S. broughtonii* was accounting for 23–26% of mitogenomes and 35–40% of URs, indicating ORFs are one of the main components of the large URs. In addition, many large ORFs (> 2000 bp) were found in Arcidae mitogenomes (Additional file 1: Table S9).

### Ka/Ks analysis for putative novel Arcidae mitochondrial ORF proteins

**Fig. 4:**
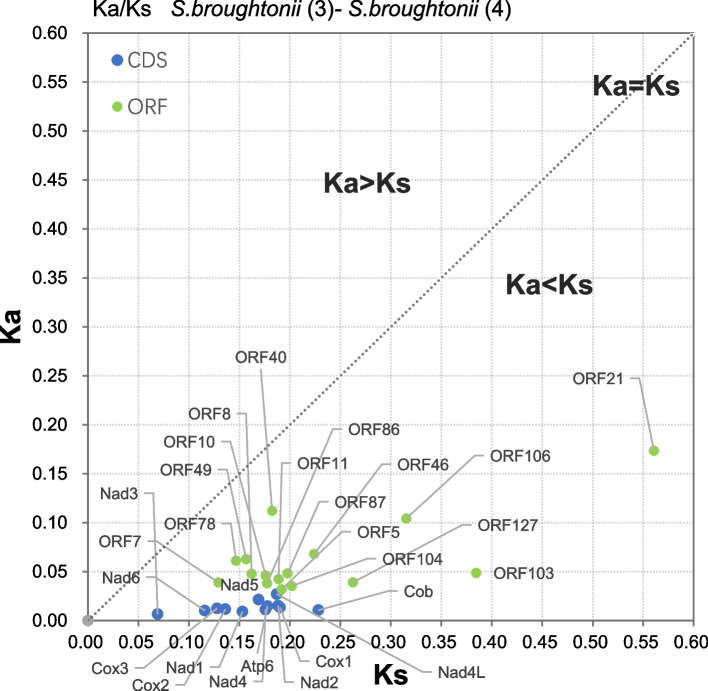
Rates of synonymous and nonsynonymous substitutions within mitochondrial protein-coding genes and ORFs identified for *Scapharca broughtonii* ((3), (4), respectively). The details are recorded in Additional file 1: Table S10. Dark blue dots indicate mitochondrial protein-coding genes. Light green dots indicate the ORFs

To estimate the degree of selection (either neutral, positive, or purifying) and genetic conservation on 12 protein coding genes and 16 ORFs, the number of nonsynonymous substitutions per nonsynonymous sites (Ka) relative to the number of synonymous substitutions per synonymous sites (Ks) was calculated. According to our results (Fig. 4 and Additional file 1: Table S10), the mean value for Ka was also different between protein-coding genes and ORFs (0.0137 vs. 0.0624; *P* < 0.001), suggesting that the ORFs accumulated more non-synonymous mutations. Twelve protein-coding genes and all ORFs were found to be under strong purifying selection (Ka/Ks < 1). The Ka/Ks of protein-coding genes was between 0.0470 (*cob*) to 0.1447 (*nad*4L). A low Ka/Ks has been a common finding for mtDNA-encoded protein genes in animals and is explained by the elimination of mildly deleterious mutations. The ORFs had a wide range of Ka/Ks ranging from 0.1267 (ORF103) to 0.615042 (ORF40), but the Ka/Ks of some ORFs (ORF5, ORF103, ORF127) were close to some protein-coding genes (*nad*4L, *nad*5). The mean value of Ka/Ks in ORFs (0.2834) was significantly higher than that of protein-coding genes (0.0874) (P < 0.001), suggesting that ORFs have been under less selective constraints than mitochondrial protein-coding genes. In addition, the level of ORF sequence conservation between mitogenomes from alignments confirmed that these ORFs have conserved regions (Additional file 4).

### Conserved secondary structures in ORF protein sequences

To assess whether mitochondrial URs possess ORFs that could have functional importance in Arcidae bivalves, we investigated the structure of these ORFs. Transmembrane (TM) helices were identified using three different programs. Twelve of 16 ORFs in *S. broughtonii* were predicted to have at least one TM-helices with both Phobius [48] and TMHMM 2.0 programs [49]. The third, TOPCONS [50], had a stricter criterion because it gives a consensus result for the protein from five different topology prediction (details see Additional file 1: Table S11). All three programs predicted two TM-helices for all ORF127 and ORF87 in different Arcidae species with 100% confidence. The number of TM-helices of other ORFs was predicted to be unstable across Arcidae species. Four or five TM-helices were found in ORF40 of different species with two software. The remaining 13 ORFs returned variable TM predictions and possessed one to four predicted transmembrane domains in different mitogenomes. In addition, signal peptide (SP) was found in the N-terminus of five ORFs (ORF40 in *S. cornea* and *S. gubernaculum*, ORF5 in *S. broughtonii* and *S. kagoshimensis*, ORF7 in *S. kagoshimensis* (1), ORF86 in *A. crebricostata*, ORF86-b in *S. inaequivalvis*, ORF87 in *S. cornea* and *S. gubernaculum*) (Additional file 1: Table S11). Notably, ORF40 of *S. cornea* and *S. gubernaculum* were predicted to have a SP and five TM-helices, and SPs are located 1–17 aa from the N’end of the predicted peptide, with a cleavage site between 17 and 18 aa. Eleven of 14 ORF5 in different mitogenomes were predicted to have a SP, and they are almost in the same position. A SP were found only in ORF7 of *S. kagoshimensis* (1), but not in other Arcidae mitogenomes. The remaining 11 ORFs were not predicted to have a SP. Moreover, some ORFs (e.g., ORF21 in *S. broughtonii* (2) (4) (5) (6), *S. cornea* and *S. gubernaculum*) were predicted to have a Rossmann fold, a tertiary fold found in proteins that bind nucleotides, such as enzyme cofactors FAD, NAD+, and NADP+ (Additional file 1: Table S12).

### Function prediction of ORF protein sequences

As these ORF sequences did not show any obvious homology with known proteins, we performed an in-depth comparative analysis using multiple programs to predict the function of ark shell mitochondrial ORFans. Results obtained for all ORFans with all programs for protein function prediction were summarized in Additional file 1: Table S13, which included the most frequent categories of hits for molecular functions, biological processes and cellular components for the mitochondrial ORFans [51, 52], and detailed motifs and domains information (HHpred, [53]; I-TASSER). Overall, the most common hits for all ORFs were proteins involved in oxidoreductase activity, nucleic acid or protein binding (e.g., helicase/hydrolase activity) and metal ion binding (e.g., nickel cation/cobalt ion binding). Some other hits were proteins with membrane association and transporter activity, for example involved in transport across membrane, establishment of protein localization, and most of all involved in intracellular transport (e.g., ORF10, ORF7, ORF104, ORF106, ORF21). Some ORF proteins pointed to a role in ATP binding, for example in cellular macromolecule metabolic process (e.g., ORF127).

In particular, most sequences analyzed returned predictions that the proteins were involved in oxidoreductase activity and metabolic process, and the predicted subcellular localizations for these ORFs were different, with some being membranes and organelles (endoplasmic reticulum and nucleus) and some being soluble outside the cell (Additional file 1: Table S14). For ORF104, the highest probability matches included proteins that have a role in obsolete oxidoreductase activity, acting on a heme group of donors, oxygen as acceptor and obsolete heme-copper terminal oxidase activity. In addition to ATP binding, ORF127 hits also included proteins related to FAD binding, nucleic acid binding and oxidase activity, which may participate in cellular processes such as cellular response to stress and nucleic acid metabolic process. Additionally, several hits of ORF11 appeared to be involved in glucan 1,4-alpha-glucosidase activity (a necessary step in the tricarboxylic acid cycle), obsolete coenzyme metabolic process and drug metabolic process. Other hits pointed to a role in cellular respiration (e.g., ORF8), one-carbon metabolic process (e.g., ORF49) and hexose metabolic process (e.g., ORF5).

Moreover, the previous study [38] proposed a viral origin for mitochondrial ORFans in DUI bivalves, therefore, we scanned our results for protein function prediction with all programs to highlight the hits related to viruses. Nine of 16 ORFs were possibly related to viral-related biological process and proteins (Additional file 1: Table S13). However, BLAST results for all ORFs (Additional file 1: Table S6) showed that most of the ORF hits are non-viral and viral-related hits have low probability (e.g., ORF7, E-value = 2.2). Hits with high probability values were bacterial or metazoan proteins (e.g., ORF40, Hypothetical protein, *Sepia pharaonic*; ORF87, MCP signaling domain protein, *Clostridium argentinense*). The same ORF in different species did not produce consistent blast result.

### Phylogenetic analyses and ancestral state reconstruction

**Fig. 5:**
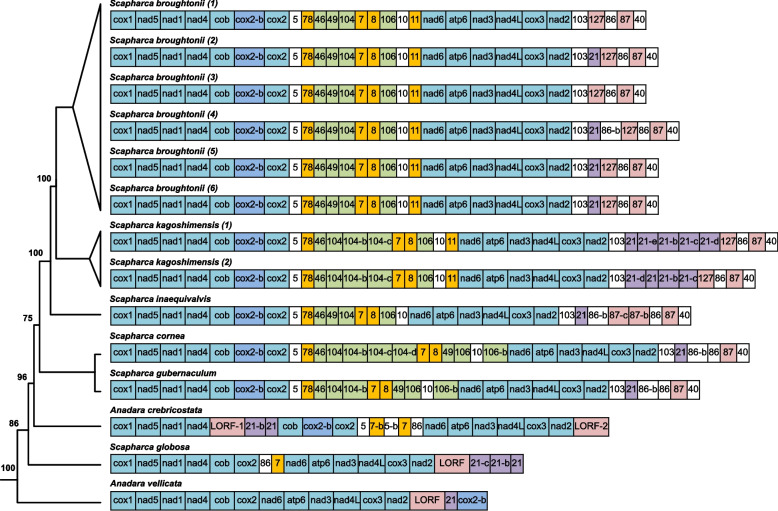
Organization of protein-coding genes and ORFs in Arcidae mitogenomes. All genes are encoded on the same strand. Genes and ORFs are denoted by standard abbreviations and are not scaled according to length. The different colored boxes indicate protein-coding genes and different ORFs (cyan: protein-coding genes; dark blue: *cox*2-b, the duplication of *cox*2, see details in Additional file 1: Table S2; orange, green, purple and pink: the ORFs that duplicate obviously; the pink LORFs: ORFs partially aligned with ORF87, see details in Additional file 1: Table S7)

**Fig. 6:**
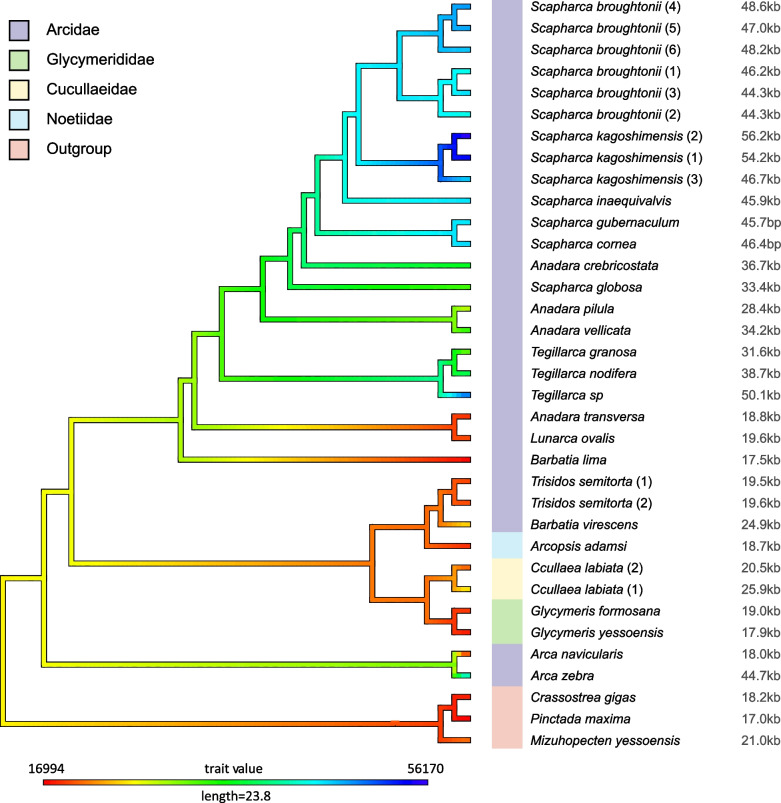
Ancestral reconstructing the evolution of mitochondrial genome size across phylogenetic tree of Arcidae. Color coding of the branches represents mitochondrial genome size with blue being larger (up to 56,170 bp) and red being shorter (16,994 bp). The different colored columns indicate different group. 23.8(kb) is a medium-sized mitogenome

The molecular phylogeny of Arcoidea was reconstructed based on the mitogenome data sets using ML (Figs. 5 and 6). After removing ambiguously aligned positions, the concatenated alignment of amino acid sequences from thirty-five taxa had a total length of 3057 positions (Table 1). Arcidae was found to be polyphyletic with three well-supported lineages. The first lineage included the subfamily Anadarinae and the sister taxon *Barbatia lima*; the second comprised two *Trisidos* species and *Barbatia virescens*; and two *Arca* species formed the third lineage. *Arcopsis adamsi*, the only representative of *Noetiidae*, was found to nest within the polyphyletic Arcidae as the sister taxon of the *Trisidos/B. virescens* clade. Within Anadarinae, *Anadara* and *Scapharca* were found to be polyphyletic. These results are consistent with previous studies [12, 44, 54–56]. Arcoidea, which includes *Arcidae*, *Noetiidae*, *Cucullaeidae* and *Glycymerididae*, formed a clade that was well-supported in the ML analysis (bootstrap support value = 100%), whereas the previous ML analysis [12] for Arcoidea was not well-supported (bootstrap support value = 56%). Ancestral state reconstruction indicated that the evolution of the mitogenome size has undergone different changes across different arcoid lineages (Fig. 6). A medium-sized mitogenome (23.8 kb) was estimated to be the ancestral state of Arcoidea. Mitogenome expansion was apparent in Anadarinae species, whereas genome contraction has occurred in *A. transversa*, *Lunarca ovalis*, *B. lima* and *Arca navicularis*. In addition, the clade, which encompasses *Trisidos* species, *B. virescens*, *Noetiidae*, *Cucullaeidae* and *Glycymerididae*, also have a slight contraction. Notably, multiple expansion takes place in genus *Scapharca*, but an independent expansion in *Arca zebra*.

## Discussion

In this study, we present multiple lines of evidence supporting the functionality of ORF at amino acid and nucleotide levels. These results support novel features for Arcidae mitochondrial genomes: the presence of additional, lineage-specific, mtDNA-encoded proteins with potential functional significance. We discuss the possible origin of ORFs including mitochondrial, nuclear and viral origins. In addition, the insertion and duplication events of these ORFs play an important role in multiple expansions of Arcidae mitogenome, which may provide significant insights on how bilaterian mitochondrial genomes evolve in terms of size variation, gene complement, and gene organization.

### Novel ORFs in Arcidae mitogenomes and their possible origins

Mitogenomes of Arcidae species with large URs have novel lineage-specific ORFs which do not show significant amino acid sequence similarity to known proteins in the database, but they have special secondary structures and meaningful hits for function prediction. The ORF amino acid sequences are conserved among different mitogenomes and found in extra-genic regions, always inside the large URs. Traditionally, URs are generally regarded as vestiges of pseudogenes generated by random deletions after gene duplication [34, 57–59], while our further examination of the two shared large URs in Arcidae reveals two categories of sequences. The first category contains sequences exhibiting many features typically associated with mitochondrial control regions, such as presence of repeat units and sequences that can form hairpin structures and stem–loop. The second category contains sequences that possess many ORFs of considerable length. Previous research [12] on the first category found significantly positive correlations between Arcidae mitochondrial genome size and proportion of tandem repeats and proportion of inverted repeats. However, the ORFs identified turned out to be one of the main components of the large URs rather than repeat families and transposable elements in our study. Some ORFs were conserved in the lineage containing Scapharca and *A. crebricostata* (Fig. 2), suggesting that they may have emerged before the speciation of *Scapharca* lineage and *A. crebricostata*. Mitochondrial URs of some ark shells are probably inserted in multiple independent evolutionary events [12], and if so these lineage-specific ORFs may arise from independent insertion events.

Previous studies [35, 38–40, 60] suggested mitochondrial ORFs could originate from different processes: (1) the duplication and subsequent modification of extant mitochondrial genes, (2) transfer of DNA from nuclear genomes to mitochondrion, (3) the insertion of viral sequences into the host mitogenome. In our study, the ORF sequences analyzed do not show homology with any known Arcidae mitochondrial protein, so they unlikely originated from recent duplication events from existing mtDNA genes. The ORF sequences we identified in *S. broughtonii* were not found in a high-quality nuclear genome of *S. broughtonii* [61] (Additional file 5), indicating they may not have been transferred from nuclear genomes. Moreover, in our analysis, hits similar to ORF sequences are mainly from proteins of bacteria, fungi, parasites and viruses (Additional file 1: Table S6 and Table S13). Since most of hits have no significant similarity or probability, they are not sufficient to determine the origin of the ORFs. The results showed that 9 of 16 ORFs in *S. broughtonii* are possibly involved in the process of viral entry into host cell and viral release from host cell, which provide clues to speculate on a possible viral origin. However, BLAST results including all Arcidae species showed that most of the ORF hits are to non-viral sequences, and the probability of some viral hits (< 60%) were low. Hits with high probability values (E-values < 0.005) are bacterial or metazoan proteins, suggesting that other organisms or other processes [62] may be the source of these ORF genes. Consequently, our results do not support a viral origin of Arcidae ORFs, although previous studies [38, 40] have suggested that some bivalve mitochondrial ORFs may originate from viruses.

In Arcidae mitogenomes, if ORFs have experienced rapid evolution since their origin, they may diverge to the extent that homology to mitochondrial proteins, nuclear sequences and viral sequences is not discernable. The ka/ks results showed that some ORF sequences contained mostly non-synonymous mutations (Additional file 1: Table S10), indicating the rapid evolution of these mitochondrial ORF genes. Fast rate of evolution may erase evidence of ORF sequence similarities (homology) among species, so we cannot fully exclude the possibility that the ORFs are derived from mitochondrial duplications, nuclear genome or even viral sequences. Of these possible origins, mitochondrial duplication interpretation is more plausible. Gene duplication is thought to be the most common mechanism underlying the origin of most novel genes [63]. In our study, large-scale duplication events (both mitochondrial genes and ORFs) were found, suggesting that duplications are prone to occur in Arcidae mitogenome. Also, this explains why many identified ORFs have TM-helices and the most common hits of them involved in metabolic process. In considering possible alternatives, these ORFs may originate from duplication of mitochondrial genes, but also call for further studies to investigate their origin.

### The insertion and duplication of mitochondrial novel ORFs and implications for UR size evolution

According to ancestral state reconstruction (Fig. 6), the mitogenome size of the common ancestor of Arcidae is relatively small (i.e., < 20 kb) like in most metazoans. This result suggests Arcidae mitogenomes have experienced multiple expansions (insertion and duplication events) with some lineages forming very large mitogenomes. For example, UR1 (regions between *cox*2 and *nad*6) is only 295 bp in the mitogenome of *A. crebricostata* (Table 2). However, we found that the UR1 of *S. globosa* mitogenome is 3197 bp, which contains the ORF sequences similar to ORF86 and ORF8. Based on phylogenetic relationships and mitogenome structure of Arcidae (Fig. 5), the inserted sequence may be the origin of the UR1 of *Scapharca* lineage and *A. crebricostata*. Subsequently, the expansion of UR1 is obvious in other *Scapharca* species (reaching above 10 kbp) (Table 2) because of the duplications of some ORFs (Fig. 5). In bivalve, many mitochondrial gene duplication events have been found such as *nad*2 duplication in the oyster genus *Crassostrea* (Bivalvia, Ostreidae) [46] and *cox*2 duplication in several DUI bivalve species [64, 65]. In our results, *S. broughtonii* mitogenomes contained a duplication of the *cox*2 gene, named *cox*2-b (Fig. 5 and Additional file 1: Table S2), a feature that has been also observed in the mitogenome of other closely related species (*Scapharca* lineage and *A. crebricostata*, except for *S. globosa*). Coincidentally, ORF duplications were found in the *Scapharca* species that have *cox*2 duplication with exception of *S. globosa*. For example, according to ORF distribution and phylogenetic relationship (Fig. 5 and Additional file 6), ORF8, ORF7, ORF11 and ORF78 (> 30% identity at the protein level) might originate from the same ORF gene and the ORF7 gene in *A. crebricostata* could have been the original copy. In addition, duplication of ORF5, ORF21, ORF104, ORF46, ORF86 and ORF87 were also found in different Arcidae mitogenomes, which suggest that a large-scale duplication event has occurred rather than just *cox*2 duplication. Meanwhile, these duplicated ORF sequences make mitogenomes larger. Most of the ORFs from duplication were over 500 bp in length, which adds at least 8217 bp to the mitogenomes of *S. broughtonii*. For example, ORF87 and its duplication of ORF127 are both over 1800 bp, which have a huge impact on mitogenome size. In conclusion, the duplication of *cox*2 and ORFs cause Arcidae mitogenomes to expand further. We think that duplication events of ORFs play an important role in multiple subsequent expansions of Arcidae mitogenome.

However, most of ORFs we identified in *S. broughtonii* are only found in *Scapharca* and *Anadara* (Fig. 2). Possible explanation is that the expansion of *Scapharca* and *Anadara* was the result of independent insertion and duplication events, which results in the ORF sequences different from other genera. To further explore expansions caused by ORFs, we reinvestigated all Arcidae mitogenomes and found 54 large ORFs (LORFs) of unknown structure and function in 18 Arcidae mitogenomes (Additional file 1: Table S9). These LORFs range in length from 933 to 5187 bp, and the average length is 2200 bp. In the mitogenomes of *Scapharca*, all LORFs were found in UR2, indicating that the emergence of LORFs may be related to UR2 expansion. Interestingly, many LORFs were also found in *Anadara* and *Tegillarca* mitogenomes, and some of them have a fragment of amino acid sequences similar to the regions of ORF87 in *Scapharca* (Fig. 2 and Additional file 1: Table S7), which implies that these LORFs might have a common origin with ORF87 in an earlier expansion event. Because of large lengths, LORFs were easily disrupted by mutations and harder to maintain. Therefore, we think that the existence of LORFs might be significative and can provide clues to explore the independent expansions of other Arcidae mitogenomes in future.

### Predicted functions for ark shell mitochondrial ORFs

In our study, there are multiple lines of evidence indicating potential functionality of 14 novel lineage-specific ORFs in *S. broughtonii*, likely as expressed proteins. In other taxa, lineage-specific ORF genes are involved in important adaptive processes and key biological functions [36, 38]. Herein, multiple lines of evidence suggest these UR ORFs are functional in nature. Transcriptome analysis indicated that the nine ORFs (Table 3 and Fig. 3) are transcribed in mitogenomes of *S. broughtonii*. Secondly, the ka/ks analysis showed that all the ORFs are under purifying selection (Fig. 4 and Additional file 1: Table S10). Thirdly, because novel ORFs do not show significant similarity to known proteins, we performed multiple analyses of their structure to predict the function. Our results for secondary structure prediction show that most of the ORFs are predicted to have functional domains (Additional file 1: Table S11), which is a significant support for identifying these ORFs as protein-coding genes. For example, we observe that four conserved TM-helices are present in ORF40 proteins of *S. broughtonii*. In addition, ORF40 returned hits to proteins with membrane association (e.g., proteins involved in tail-anchored membrane protein insertion into ER membrane), and the predicted subcellular localizations with DeepLoc for ORF40 are also endoplasmic reticulum and membrane. The ORF127 protein is very long in length and has two stable TM-helices among different Arcidae species. Our prediction results, together with transcriptome and ka/ks analysis, showed that these lineage-specific ORF proteins that occur in Arcidae mitogenomes may have underlying functions. For example, ORF21 and ORF103 may be candidates for *atp8*, which has not been annotated in many bivalves [6] and is not reported in Arcidae. The two ORFs are approximately the same length as *atp8* and are transcribed in *S. broughtonii*. They are under strong purifying selection and their relative solvent accessibility is similar to the *atp8* gene (Additional file 7). ORF21 is conserved at the amino acid level and has a broader distribution than ORF103 (Fig. 2). ORF103 is predicted to have TM-helices, but ORF21 does not. Compared to ORF21, we believe that ORF103 is a more likely candidate for *atp8* because the TM-helix is important for *atp8* function. However, ORF21 and ORF103 do not show homology with any bivalve atp8 genes. Therefore, we cannot determine if either of these two ORFs is an *atp8* gene homolog.

Moreover, based on the functional prediction analysis, we speculated that some of novel ORF proteins in mitochondrial URs of Arcidae may have acquired new functions. A previous study showed that ORFans in bivalves with DUI may have a viral origin and be involved in the maintenance of sperm mitochondria during embryo development [38, 40]. Our results for molecular function prediction show that the ORFs have different functions hits, the most common hits involved in metabolic process (e.g., ATP association activity, oxidoreductase activity, glucan 1,4-alpha-glucosidase activity). Several Arcidae species are limited locomotive and more tolerant to hypoxia, such as S. kagoshimensis, which lead to low metabolic rate [43, 66, 67]. The large mitogenome size in these bivalves may be correlated with their metabolic rates because the relaxed selective constraints of mitogenomes may be energy-related [12, 68]. In addition, genes participating in ATP and lipid metabolism under selection were found to be important in thermal adaptation for oysters [69]. Lineage-specific genes could participate more in lineage-specific adaptation [70], therefore, we speculated that the functions of lineage-specific ORF genes are related to low metabolic rate or thermal adaptation, which may provide new insights into the function of large URs in Arcidae mitogenomes.

Finally, the possibility that ORFs may be pseudogenes and do not perform any function cannot be ruled out. According to the results, some ORFs are unstable in the number of TM-helices such as ORF104, ORF106 and ORF46. The number of TM-helices in a given ORF vary among Arcidae species, indicating that these ORFs are not conserved at the secondary structure level. The results suggest a possibility that ORFs may have different adaptations in various Arcidae species. Another possibility is that the ORFs could be pseudogenes or in the process of pseudogenization after insertion and duplication events. In many cases duplicated genes are subject to pseudogenization [1], which appears to be the most likely fate for mitochondrial gene duplications. But compared to pseudogenes, the ORFs we identified are more conserved at the nucleotide sequence level and some of them may be transcriptionally active, so they are likely to be in the process of pseudogenization but more research is needed.

### Dynamic changes in intraspecific mitogenome size

Different mitogenomes in the same Arcidae species vary dramatically in length. The previous studies have demonstrated that tandem repeats are potentially a main factor leading to variation of intraspecific mitogenome size [12, 16]. For example, mitogenomes of *S. broughtonii* ranges in size from about 47 kb to ∼ 50 kb due to variation in the number of tandem repeats [13]. The four *S. broughtonii* mitogenomes we assembled also have many tandem repeats (Additional file 1: Table S3). However, variation of these tandem repeat size does not fully explain the variation (44,327–48,560 bp) of the URs of *S. broughtonii* mitogenomes because the latter is larger. By observing URs length (Table 2), UR1 in *S. broughtonii* and *S. kagoshimensis* are almost the same length, and UR2 is highly variable and responsible for the variation of mitogenomes in length. We propose that variation of the length of *S. broughtonii* and *S. kagoshimensis* mitogenomes may be caused by incomplete assembly of the UR2 because of their complex content. Large-scale repeat sequences are difficult to sequence using conventional Sanger and short-read sequencing methods [71]. Theoretically, repeats extend beyond read length, mitogenome assemblies are limited within the boundaries of repetitive elements [72]. Although long-range PCR can be used to amplify DNA regions of several kilobases, sequencing through repetitive regions often results in ambiguous and/or erroneous sequence reads as a consequence of self-priming of randomly-amplified repeat-segments, chimeras and/or jumping PCR artefacts [73, 74]. For example, the *S. kagoshimensis* mitogenome (KF750628) were assembled using long-PCR into a circle [18], but some protein-coding genes and ORFs are fragmented, which suggest a low-quality assembly. Hence, future studies can focus on long-read sequencing for Arcidae mitogenome assembly, which can achieve read lengths of 80 kb to > 1 Mb, enabling repetitive and structurally complex DNA elements to be resolved with confidence [75, 76].

## Conclusions

In this study, we found 14 special ORFs in the large unassigned regions in mitogenomes of *S. broughtonii*. Interestingly, these putative additional proteins have also been found in other species of genus *Scapharca*. We present multiple lines of evidence supporting the functionality of ORFs at amino acid and nucleotide levels and discuss their possible origin. These results support novel features for Arcidae mitochondrial genomes: the presence of lineage-specific mitochondrial ORFs with transcriptional activity and potential functional significance. Moreover, our study reveals that the insertion and duplication events of ORFs play an important role in multiple expansions of Arcidae mitogenome. Although other bilaterian taxa have expansion regions in their mitochondrial genome, those in Arcidae are most extreme. Thus, Arcidae may provide significant insights on how bilaterian mitochondrial genomes evolve in terms of size variation, gene complement, and gene organization.

## Methods

### Specimen collection and sequencing

Adult *S. broughtonii* specimens were obtained from populations near Qingdao (the tuandao market), Shandong Province, China. One specimen of *S. cornea*, was collected from a local market in the Philippines. After collection, all specimens were immediately preserved at − 80 °C or in 95% ethanol. Species were identified using morphology and genetic distance of Arcidae mitochondrial *Cox1*. Total genomic DNA of four *S. broughtonii* and one *S. cornea* individuals was extracted from adductor muscle using the TIANamp Marine Animals DNA Kit according to the manufacturer’s instructions and sequenced on an Illumina HiSeq X using 2 × 150 paired-end (PE) library. Total mRNA extraction of six *S. broughtonii* individuals was performed from adductor muscle using a Trizol protocol. All procedures were carried out on ice and quickly to avoid RNA degeneration. The extracted mRNA was sequenced with a paired-end (PE) library with an insert size of 250 bp. The sequencing of genomic DNA and RNA was both performed by Tianjin Novogene Bioinformatics Technology Co., Ltd., China.

### Mitochondrial genome assemblies and annotation

Raw data from four *S. broughtonii*, one *S. kagoshimensis* (SRX8857271) and one *S. cornea* individuals were filtered using Trimmomatic 0.39 [77] for removal of TruSeq adapter sequence and trimming low-quality bases from the ends of each read. Clean short reads were assembled de novo using Novoplasty 4.0 [78], Mitoz v2.3 [79] with the all module and SPAdes v3.11.1 [80] with k-mer of 21, 33, 55, 77 and the –careful flag, respectively. Then, assembly results were searched using BLAST [47] against a nucleotide database constructed from the complete mitogenome of *S. broughtonii* (AB729113) to find the mitochondrial contigs. Some partial mtDNA contigs were recovered in the assembly results of SPAdes. In order to bridge contigs together into a single contig, Price1.0 [81] was applied to extend and join these partial contigs with default settings by iteratively adding sequence reads to the contig ends. Finally, mitogenomes from different assemblers were assessed using Quast 5.0 [82] based on genome fraction, total aligned length, duplication ratio, and level of completeness. The above programs were installed on Linux system via Bioconda [83]. All the newly sequenced mitogenome sequences have already been deposited in GenBank, and the accession numbers are listed in Table 1.

Locations of the protein-coding genes (PCGs), transfer RNAs (tRNAs) and ribosomal RNAs (rRNAs) were determined initially with the MITOS web server using the invertebrate mitochondrial genetic code and validated using MFannot [84]. ORF Finder (https://www.ncbi.nlm.nih.gov/orffinder/) and BLAST were employed to examine and adjust manually gene boundaries by comparison with the published Arcidae mitochondrial genes since MITOS seems to underestimate gene length. The visualization of mitogenomes was performed using CGView [85]. Large URs structure was defined using blastn with manual alignments. ORFs in large URs of six mitogenomes were identified with ORF Finder using the invertebrate mitochondrial genetic code.

### Transcriptome analysis

Quality assessment of RNA reads from six *S. broughtonii* individuals was carried out using FastQC v0.11.9 [86]. Trimmomatic 0.39 was employed for removal of adaptor sequences and low-quality positions. Then HISAT2 [87] was used to align clean reads from each individual to the reference mitogenomes that had been newly assembled. The mapped reads were sorted, indexed using Samtools [88] and were assembled using Stringtie [89] in a reference-based approach. Next, we used BLAST to search the StringTie gene sets against the database of UniProtKB/Swiss-Prot proteins [90], and identified the protein domain with PFAM [91]. TransDecoder v5.5.0 was used to predict ORFs (> 20 aa) that had coding potential from assembled transcripts. The results from BLASTP and hmmscan [92] searches were used to inform the final TransDecoder prediction step.

To estimate expression levels, we summarized information on annotated mitochondrial PCGs and predicted ORFs and used Artemis [93] to make a GTF file (*S. broughtonii* (4) as a reference mitogenome). FeatureCounts v2.0.1 [94] was used to count mapped reads on BAM files with the option “--primary, -t exon, -g gene_id”. All other parameter values were left at their defaults. BAM files were from the HISAT2 results of three *S. broughtonii* transcriptomes, which are from the same experimental batch. Finally, we used a R pipeline (Additional file 2) to estimate expression levels in TPM for each mitochondrial PCGs and ORFs. Heatmap and boxplot were made with R package pheatmap and ggplot2 [95].

### Novel mitochondrial ORFs and sequence conservation

To assess the presence of novel ORFs in other Arcidae mitogenomes in which they were not annotated, and at the same time validate annotations, we used BLAST to align with E-values < 0.00001 and ORF Finder to search for all possible ORFs ≥75 nucleotides long under the invertebrate mitochondrial genetic code from the 32 Arcidae mitogenomes, and then translated them into the corresponding proteins. The duplicated ORFs were classified based on BLAST results and molecular phylogeny. Because ORF protein sequences vary little within the same species, only one sequence was used for analyzing. For comparative purposes, alignments of the putative novel mitochondrial proteins in different Arcidae species were run with MAFFT v7.475 [96]. Finally, we used Jalview [97] to visualize the alignments. Then we used a ML method implemented in KaKs_Calculatorv 2.0 [98] to estimate ratios of nonsynonymous and synonymous substitution rates (Ka/Ks) in mitochondrial PCGs and ORFs between sister pairs of *S. broughtonii*. These comparisons facilitated a better understanding of the selection pressures acting on protein coding genes and ORFs. ORF names were given according to the result of ORF Finder in *S. broughtonii*.

### Protein structural and functional analysis

The above-mentioned ORFs were translated and analyzed at the amino acid level. Putative transmembrane (TM) helices were identified using a variety of protein signature recognition methods implemented by the following programs: Phobius [48], TMHMM 2.0 [49] and TOPCONS [50]. Evidence of signal peptides (SPs) was sought using Phobius, TOPCONS, SignalP 4.0 [99] and TargetP 2.0 [100]. HHpred [53] were used to search for known functional sequence motifs and domains. Cofactory 1.0 [101] was used to identify Rossmann fold sequence domains and predicts their specificity for the cofactors FAD, NAD or NADP. Subcellular localizations (e.g., cell membrane, cytoplasm, nucleus, etc.) were predicted using DeepLoc-1.0 [102] and Euk-mPLoc 2.0 (Cell-PLoc 2.0 package) [103].

The following procedures were used to predict the function of ORF proteins: (1) we performed BLASTp, tBLASTx, and PSI-BLAST searches against NCBI entire non-redundant protein database (NRDB) with default parameters [47] and BLAST searches against UniProt (UniProtKB reference proteomes + Swiss-Prot) with default parameters. (2) we used HHpred for profile HMM – profile HMM comparisons, which compares HMM profiles with databases of HMMs representing proteins with annotated protein families (e.g., PFAM, SMART, CDD, COGs, KOGs) or known structure (e.g., PDB, SCOP). (2) I-TASSER, which uses a hierarchical protein structure modeling approach that is based on the secondary-structure enhanced profile–profile threading alignment to predict protein tertiary structure and function [52]. (3) PredictProtein, which predicts aspects of protein structure (secondary structure, solvent accessibility, transmembrane helices and strands, coiled-coil regions, disulfide bonds and disordered regions) and function (identification of functional regions, homology-based inference of Gene Ontology terms, comprehensive subcellular localization prediction, protein-protein binding sites, protein-polynucleotide binding sites and predictions of the effect of point mutations [non-synonymous SNPs] on protein function) [51]. For HHpred, I-TASSER and PredictProtein, only top three results were recorded.

### Phylogenetic analysis and ancestral state reconstruction

We used 12 mitochondrial PCGs to reconstruct the phylogenetic history of 35 mitogenomes: six new assemblies and twenty-six published assemblies of Arcidae and outgroup species. Thirty-five taxa representing 26 species were included in the phylogenetic analyses and presented in Table 1. For phylogenetic analyses, outgroup species *Mizuhopecten yessoensis* [45] from Pectinidae, *Pinctada maxima* from Pteriidae, *Crassostrea gigas* from Ostreidae were selected based on data availability and bivalve evolutionary history. We extracted all protein coding gene sequences, except *atp*8, from the 35 mitogenomes and translated them with the invertebrate mitochondrial genetic code. Then amino acid sequences of these genes were aligned by Mafft 7.475 and then ambiguously aligned regions were removed in TrimAl 1.4 [104] under the “automated1” setting. The resultant alignments were subsequently concatenated using SeqKit [105] and used for maximum-likelihood (ML) phylogenetic inference with IQ-TREE v2.0.3 [106]. An optimal substitution model was automatically selected, whose robustness was assessed with 1000 replicates of ultrafast bootstrapping. The generated tree was depicted and submitted to FigTree v.1.4.4 for visualization and annotation. Ancestral states for mitogenome size were reconstructed using the ‘fastAnc’ function (fast ML estimation) under a Brownian motion mode and visualised on the tree with the ‘contMap’ function, both from the R package Phytools v.0.7–70 [107]. Arcidae sequences are highly variable in length within species, and so multiple mitogenomes were analyzed per species to provide a more complete picture of intraspecific mitogenome size evolution.

## Supplementary Information

**Additional file 1.** Table S1-S4. Table S5. Table S6. Table S7-S12 and S14. Table S13.

**Additional file 2.** R pipeline and TPM file.

**Additional file 3.** All ORF sequences.

**Additional file 4.** ORF sequence alignment.

**Additional file 5 **The Blastn and Blastx results of *Scapharca broughtonii* ORFs with nuclear genome.

**Additional file 6.** The tree of duplicated ORFs.

**Additional file 7.** Secondary structure and relative solvent accessibility of ATP8 and ORFs.

## Data Availability

The data underlying this article are available in the article and in its Supplementary Material as well as in the GenBank Nucleotide Database and can be accessed with OM807131- OM807136. Sequencing data files are available through the NCBI Sequence Read Archive (BioProject: PRJNA809524).

## Abbreviations

aa: Amino acid
atp8: ATP synthase F0 subunit 8 gene
cox2: Cytochrome c oxidase subunit 2 gene
DUI: Doubly uniparental inheritance (of mitochondria)
HMM: Hidden Markov Model
LORFs: Large ORFs
mitogenome: Mitochondrial genome
ML: Maximum likelihood
mtDNA: Mitochondrial DNA
nad2, − 4 L, − 5, 6: NADH dehydrogenase subunits 2, 4 L, 5, 6 genes, respectively
N-terminus: Start of a protein chain
ORF: Open reading frame
ORFan: ORF with no recognizable homology or similarity to known genes
tRNA: Transfer RNA
unassigned regions: Regions that are functionally unassigned
URs: Unassigned regions

## References

[1] Breton S, Milani L, Ghiselli F, Guerra D, Stewart DT, Passamonti M (2014). A resourceful genome: updating the functional repertoire and evolutionary role of animal mitochondrial DNAs. Trends Genet.

[2] Roger AJ, Muñoz-Gómez SA, Kamikawa R (2017). The origin and diversification of mitochondria. Curr Biol.

[3] Andersson SG, Kurland CG (1998). Reductive evolution of resident genomes. Trends Microbiol.

[4] Embley TM, Martin W (2006). Eukaryotic evolution, changes and challenges. Nature..

[5] Boore JL (1999). Animal mitochondrial genomes. Nucleic Acids Res.

[6] Gissi C, Iannelli F, Pesole G (2008). Evolution of the mitochondrial genome of Metazoa as exemplified by comparison of congeneric species. Heredity..

[7] Vallès Y, Halanych KM, Boore JL (2008). Group II introns break new boundaries: presence in a bilaterian's genome. PLoS One.

[8] Zardoya R (2020). Recent advances in understanding mitochondrial genome diversity. F1000Research..

[9] Lindberg WP (2008). Phylogeny and evolution of the Mollusca.

[10] Ghiselli F, Gomes-dos-Santos A, Adema CM, Lopes-Lima M, Sharbrough J, Boore JL (2021). Molluscan mitochondrial genomes break the rules. Philos T R Soc B.

[11] Morton BS, Prezant RS, Wilson B, Beesley PL, Ross GJB, Wells A (1998). Class Bivalvia. Mollusca: the southern synthesis.

[12] Kong L, Li Y, Kocot KM, Yang Y, Qi L, Li Q (2020). Mitogenomics reveals phylogenetic relationships of Arcoida (Mollusca, Bivalvia) and multiple independent expansions and contractions in mitochondrial genome size. Mol Phylogenet Evol.

[13] Liu Y, Kurokawa T, Sekino M, Tanabe T, Watanabe K (2013). Complete mitochondrial DNA sequence of the ark shell Scapharca broughtonii: an ultra-large metazoan mitochondrial genome. Comparative Biochemistry and Physiology Part D: Genomics and Proteomics.

[14] Hou Y, Wu B, Liu Z, Yang A, Ren J, Zhou L (2016). Complete mitochondrial genome of ark shell Scapharca subcrenata. Mitochondrial DNA Part A.

[15] Sun S, Li Q, Kong L, Yu H (2020). Evolution of mitochondrial gene arrangements in Arcidae (Bivalvia: Arcida) and their phylogenetic implications. Mol Phylogenet Evol.

[16] Smith DR, Snyder M (2007). Complete mitochondrial DNA sequence of the scallop Placopecten magellanicus: evidence of transposition leading to an uncharacteristically large mitochondrial genome. J Mol Evol.

[17] Williams S, Foster P, Hughes C, Harper E, Taylor J, Littlewood D (2017). Curious bivalves: systematic utility and unusual properties of anomalodesmatan mitochondrial genomes. Mol Phylogenet Evol.

[18] Sun S, Kong L, Yu H, Li Q (2015). Complete mitochondrial genome of Anadara vellicata (Bivalvia: Arcidae): a unique gene order and large atypical non-coding region. Comparative Biochemistry and Physiology Part D: Genomics and Proteomics..

[19] Ghiselli F, Milani L, Guerra D, Chang PL, Breton S, Nuzhdin SV (2013). Structure, transcription, and variability of metazoan mitochondrial genome: perspectives from an unusual mitochondrial inheritance system. Genome biology and evolution.

[20] Lynch M, Koskella B, Schaack S (2006). Mutation pressure and the evolution of organelle genomic architecture. Science..

[21] Spinelli JB, Haigis MC (2018). The multifaceted contributions of mitochondria to cellular metabolism. Nat Cell Biol.

[22] Pont-Kingdon GA, Okada NA, Macfarlane JL, Beagley CT, Wolstenholme DR, Cavalier-Smith T (1995). A coral mitochondrial mutS gene. Nature..

[23] Bilewitch JP, Degnan SM (2011). A unique horizontal gene transfer event has provided the octocoral mitochondrial genome with an active mismatch repair gene that has potential for an unusual self-contained function. BMC Evol Biol.

[24] Ogata H, Ray J, Toyoda K, Sandaa R-A, Nagasaki K, Bratbak G (2011). Two new subfamilies of DNA mismatch repair proteins (MutS) specifically abundant in the marine environment. The ISME journal.

[25] Pett W, Lavrov DV (2013). The twin-arginine subunit C in Oscarella: origin, evolution, and potential functional significance. Integr Comp Biol.

[26] Shao Z, Graf S, Chaga OY, Lavrov DV (2006). Mitochondrial genome of the moon jelly Aurelia aurita (Cnidaria, Scyphozoa): a linear DNA molecule encoding a putative DNA-dependent DNA polymerase. Gene..

[27] Kienzle L, Bettinazzi S, Brunet M, Choquette T, Khorami HH, Roucou X, Landry CR, Angers A, Breton S (2022). MTALTND4, a second protein coded by nd4 impacts mitochondrial bioenergetics.

[28] Lee C, Zeng J, Drew BG, Sallam T, Martin-Montalvo A, Wan J (2015). The mitochondrial-derived peptide MOTS-c promotes metabolic homeostasis and reduces obesity and insulin resistance. Cell Metab.

[29] Cobb LJ, Lee C, Xiao J, Yen K, Wong RG, Nakamura HK (2016). Naturally occurring mitochondrial-derived peptides are age-dependent regulators of apoptosis, insulin sensitivity, and inflammatory markers. Aging (Albany NY).

[30] Miller B, Kim SJ, Kumagai H, Mehta HH, Xiang W, Liu J (2020). Peptides derived from small mitochondrial open reading frames: genomic, biological, and therapeutic implications. Exp Cell Res.

[31] Cohen P (2014). New role for the mitochondrial peptide humanin: protective agent against chemotherapy-induced side effects. J Natl Cancer Inst.

[32] Lee C, Yen K, Cohen P (2013). Humanin: a harbinger of mitochondrial-derived peptides?. Trends in Endocrinology & Metabolism.

[33] Zhan X, Zhang S, Gu Z, Wang A (2018). Complete mitochondrial genomes of two pearl oyster species (Bivalvia: Pteriomorphia) reveal novel gene arrangements. J Shellfish Res.

[34] Breton S, Beaupré HD, Stewart DT, Piontkivska H, Karmakar M, Bogan AE (2009). Comparative mitochondrial genomics of freshwater mussels (Bivalvia: Unionoida) with doubly uniparental inheritance of mtDNA: gender-specific open reading frames and putative origins of replication. Genetics..

[35] Breton S, Ghiselli F, Passamonti M, Milani L, Stewart DT, Hoeh WR (2011). Evidence for a fourteenth mtDNA-encoded protein in the female-transmitted mtDNA of marine mussels (Bivalvia: Mytilidae). PLoS One.

[36] Breton S, Stewart DT, Shepardson S, Trdan RJ, Bogan AE, Chapman EG (2011). Novel protein genes in animal mtDNA: a new sex determination system in freshwater mussels (Bivalvia: Unionoida)?. Mol Biol Evol.

[37] Milani L, Ghiselli F (2015). Mitochondrial activity in gametes and transmission of viable mtDNA. Biol Direct.

[38] Milani L, Ghiselli F, Guerra D, Breton S, Passamonti M (2013). A comparative analysis of mitochondrial ORFans: new clues on their origin and role in species with doubly uniparental inheritance of mitochondria. Genome Biology and Evolution.

[39] Milani L, Ghiselli F, Maurizii MG, Nuzhdin SV, Passamonti M (2014). Paternally transmitted mitochondria express a new gene of potential viral origin. Genome biology and evolution..

[40] Mitchell A, Guerra D, Stewart D, Breton S (2016). In silico analyses of mitochondrial ORFans in freshwater mussels (Bivalvia: Unionoida) provide a framework for future studies of their origin and function. BMC Genomics.

[41] Sun S, Kong L, Yu H, Li Q (2015). The complete mitochondrial DNA of Tegillarca granosa and comparative mitogenomic analyses of three Arcidae species. Gene..

[42] Sun S, Kong L, Yu H, Li Q (2015). The complete mitochondrial genome of Scapharca kagoshimensis (Bivalvia: Arcidae). Mitochondrial DNA.

[43] Sun S, Li Q, Kong L, Yu H (2017). Limited locomotive ability relaxed selective constraints on molluscs mitochondrial genomes. Sci Rep.

[44] Feng Y, Li Q, Kong L (2015). Molecular phylogeny of Arcoidea with emphasis on Arcidae species (Bivalvia: Pteriomorphia) along the coast of China: challenges to current classification of arcoids. Mol Phylogenet Evol.

[45] Wu X, Xu X, Yu Z, Kong X. Comparative mitogenomic analyses of three scallops (Bivalvia: Pectinidae) reveal high level variation of genomic organization and a diversity of transfer RNA gene sets. BMC Res Notes. 2009;2(1):69. 10.1186/1756-0500-2-69 PMC2683862 19416513

[46] Wu X, Li X, Li L, Xu X, Xia J, Yu Z (2012). New features of Asian Crassostrea oyster mitochondrial genomes: a novel alloacceptor tRNA gene recruitment and two novel ORFs. Gene..

[47] Altschul SF, Madden TL, Schäffer AA, Zhang J, Zhang Z, Miller W (1997). Gapped BLAST and PSI-BLAST: a new generation of protein database search programs. Nucleic Acids Res.

[48] Käll L, Krogh A, Sonnhammer EL (2007). Advantages of combined transmembrane topology and signal peptide prediction—the Phobius web server. Nucleic Acids Res.

[49] Krogh A, Larsson B, Von Heijne G, Sonnhammer EL (2001). Predicting transmembrane protein topology with a hidden Markov model: application to complete genomes. J Mol Biol.

[50] Bernsel A, Viklund H, Hennerdal A, Elofsson A (2009). TOPCONS: consensus prediction of membrane protein topology. Nucleic Acids Res.

[51] Rost B, Yachdav G, Liu J (2004). The predictprotein server. Nucleic Acids Res.

[52] Zhang Y (2008). I-TASSER server for protein 3D structure prediction. BMC bioinformatics..

[53] Söding J, Biegert A, Lupas AN (2005). The HHpred interactive server for protein homology detection and structure prediction. Nucleic Acids Res.

[54] Combosch DJ, Giribet G (2016). Clarifying phylogenetic relationships and the evolutionary history of the bivalve order Arcida (Mollusca: Bivalvia: Pteriomorphia). Mol Phylogenet Evol.

[55] Marko PB (2002). Fossil calibration of molecular clocks and the divergence times of geminate species pairs separated by the isthmus of Panama. Mol Biol Evol.

[56] Matsumoto M (2003). Phylogenetic analysis of the subclass Pteriomorphia (Bivalvia) from mtDNA COI sequences. Mol Phylogenet Evol.

[57] Akasaki T, Nikaido M, Tsuchiya K, Segawa S, Hasegawa M, Okada N (2006). Extensive mitochondrial gene arrangements in coleoid Cephalopoda and their phylogenetic implications. Mol Phylogenet Evol.

[58] Boore JL (2006). The complete sequence of the mitochondrial genome of Nautilus macromphalus (Mollusca: Cephalopoda). BMC Genomics.

[59] Serb JM, Lydeard C (2003). Complete mtDNA sequence of the north American freshwater mussel, Lampsilis ornata (Unionidae): an examination of the evolution and phylogenetic utility of mitochondrial genome organization in Bivalvia (Mollusca). Mol Biol Evol.

[60] Burger G, Gray MW, Lang BF (2003). Mitochondrial genomes: anything goes. Trends Genet.

[61] Bai C, Xin L, Rosani U, Wu B, Wang Q, Duan X (2019). Chromosomal-level assembly of the blood clam, Scapharca (Anadara) broughtonii, using long sequence reads and hi-C. GigaScience..

[62] Tautz D, Domazet-Lošo T (2011). The evolutionary origin of orphan genes. Nat Rev Genet.

[63] Kaessmann H (2010). Origins, evolution, and phenotypic impact of new genes. Genome Res.

[64] Passamonti M, Ricci A, Milani L, Ghiselli F (2011). Mitochondrial genomes and doubly uniparental inheritance: new insights from Musculista senhousia sex-linked mitochondrial DNAs (Bivalvia Mytilidae). BMC Genomics.

[65] Passamonti M, Scali V (2001). Gender-associated mitochondrial DNA heteroplasmy in the venerid clam Tapes philippinarum (Mollusca Bivalvia). Curr Genet.

[66] Chong RA, Mueller RL (2013). Low metabolic rates in salamanders are correlated with weak selective constraints on mitochondrial genes. Evolution: international journal of organic. Evolution..

[67] Sun S, Li Q, Kong L (2021). Relaxation of selective constraint on the ultra-large mitochondrial genomes of Arcidae (Mollusca: Bivalvia). J Ocean Univ China.

[68] Strotz LC, Saupe EE, Kimmig J, Lieberman BS (1885). Metabolic rates, climate and macroevolution: a case study using Neogene molluscs. Proc R Soc B.

[69] Li A, Li L, Zhang Z, Li S, Wang W, Guo X (2021). Noncoding variation and transcriptional plasticity promote thermal adaptation in oysters by altering energy metabolism. Mol Biol Evol.

[70] Cai JJ, Petrov DA (2010). Relaxed purifying selection and possibly high rate of adaptation in primate lineage-specific genes. Genome biology and evolution..

[71] Tørresen OK, Star B, Mier P, Andrade-Navarro MA, Bateman A, Jarnot P (2019). Tandem repeats lead to sequence assembly errors and impose multi-level challenges for genome and protein databases. Nucleic Acids Res.

[72] Formenti G, Rhie A, Balacco J, Haase B, Mountcastle J, Fedrigo O (2021). Complete vertebrate mitogenomes reveal widespread repeats and gene duplications. Genome Biol.

[73] Hommelsheim CM, Frantzeskakis L, Huang M, Ülker B (2014). PCR amplification of repetitive DNA: a limitation to genome editing technologies and many other applications. Sci Rep.

[74] Hu M, Jex AR, Campbell BE, Gasser RB (2007). Long PCR amplification of the entire mitochondrial genome from individual helminths for direct sequencing. Nat Protoc.

[75] Kono N, Arakawa K (2019). Nanopore sequencing: review of potential applications in functional genomics. Develop Growth Differ.

[76] Van-Dijk EL, Jaszczyszyn Y, Naquin D, Thermes C (2018). The third revolution in sequencing technology. Trends Genet.

[77] Bolger AM, Lohse M, Usadel B (2014). Trimmomatic: a flexible trimmer for Illumina sequence data. Bioinformatics..

[78] Dierckxsens N, Mardulyn P, Smits G (2017). NOVOPlasty: de novo assembly of organelle genomes from whole genome data. Nucleic Acids Res.

[79] Meng G, Li Y, Yang C, Liu S (2019). MitoZ: a toolkit for animal mitochondrial genome assembly, annotation and visualization. Nucleic Acids Res.

[80] Bankevich A, Nurk S, Antipov D, Gurevich AA, Dvorkin M, Kulikov AS (2012). SPAdes: a new genome assembly algorithm and its applications to single-cell sequencing. J Comput Biol.

[81] Ruby JG, Bellare P, DeRisi JL (2013). PRICE: software for the targeted assembly of components of (Meta) genomic sequence data. G3: genes, genomes. Genetics..

[82] Gurevich A, Saveliev V, Vyahhi N, Tesler G (2013). QUAST: quality assessment tool for genome assemblies. Bioinformatics..

[83] Grüning B, Dale R, Sjödin A, Chapman BA, Rowe J, Tomkins-Tinch CH (2018). Bioconda: sustainable and comprehensive software distribution for the life sciences. Nat Methods.

[84] Beck N, Lang B (2010). MFannot, organelle genome annotation websever.

[85] Grant JR, Stothard P (2008). The CGView server: a comparative genomics tool for circular genomes. Nucleic Acids Res.

[86] Andrews S. FastQC. A qual control tool for high throughput sequence data. 2010. http://www.bioinformatics.babraham.ac.uk/projects/fastqc/.

[87] Kim D, Paggi JM, Park C, Bennett C, Salzberg SL (2019). Graph-based genome alignment and genotyping with HISAT2 and HISAT-genotype. Nat Biotechnol.

[88] Li H, Handsaker B, Wysoker A, Fennell T, Ruan J, Homer N (2009). The sequence alignment/map format and SAMtools. Bioinformatics..

[89] Pertea M, Pertea GM, Antonescu CM, Chang TC, Mendell JT, Salzberg SL (2015). StringTie enables improved reconstruction of a transcriptome from RNA-seq reads. Nat Biotechnol.

[90] Consortium U (2015). UniProt: a hub for protein information. Nucleic Acids Res.

[91] El-Gebali S, Mistry J, Bateman A, Eddy SR, Luciani A, Potter SC (2019). The Pfam protein families database in 2019. Nucleic Acids Res.

[92] Johnson LS, Eddy SR, Portugaly E (2010). Hidden Markov model speed heuristic and iterative HMM search procedure. BMC bioinformatics.

[93] Rutherford K, Parkhill J, Crook J, Horsnell T, Rice P, Rajandream MA (2000). Artemis: sequence visualization and annotation. Bioinformatics..

[94] Liao Y, Smyth GK, Shi W (2014). featureCounts: an efficient general purpose program for assigning sequence reads to genomic features. Bioinformatics..

[95] Wickham H (2011). ggplot2. Wiley interdisciplinary reviews: computational statistics.

[96] Katoh K, Standley DM (2013). MAFFT multiple sequence alignment software version 7: improvements in performance and usability. Mol Biol Evol.

[97] Waterhouse AM, Procter JB, Martin DM, Clamp M, Barton GJ (2009). Jalview version 2—a multiple sequence alignment editor and analysis workbench. Bioinformatics..

[98] Wang S, Huang Y, Liu S, Lin Z, Zhang Y, Bao Y (2021). Hemoglobins from Scapharca subcrenata (Bivalvia: Arcidae) likely play an bactericidal role through their peroxidase activity. Comparative biochemistry and physiology Part B, Biochemistry & molecular biology.

[99] Petersen TN, Brunak S, Von Heijne G, Nielsen H (2011). SignalP 4.0: discriminating signal peptides from transmembrane regions. Nat Methods.

[100] Emanuelsson O, Nielsen H, Brunak S, Von Heijne G (2000). Predicting subcellular localization of proteins based on their N-terminal amino acid sequence. J Mol Biol.

[101] Geertz-Hansen HM, Blom N, Feist AM, Brunak S, Petersen TN (2014). Cofactory: sequence-based prediction of cofactor specificity of Rossmann folds. Proteins: Structure, Function, and Bioinformatics.

[102] Almagro Armenteros JJ, Sønderby CK, Sønderby SK, Nielsen H, Winther O (2017). DeepLoc: prediction of protein subcellular localization using deep learning. Bioinformatics..

[103] Chou KC, Shen HB (2010). Cell-PLoc 2.0: an improved package of web-servers for predicting subcellular localization of proteins in various organisms. Nat Sci.

[104] Capella-Gutiérrez S, Silla-Martínez JM, Gabaldón T. trimAl: a tool for automated alignment trimming in large-scale phylogenetic analyses. Bioinformatics. 2009;25(15):1972–73. 10.1093/bioinformatics/btp348 PMC2712344 19505945

[105] Shen W, Le S, Li Y, Hu F. SeqKit: a cross-platform and ultrafast toolkit for FASTA/Q file manipulation. PloS One. 2016;11(10):e0163962. 10.1371/journal.pone.0163962 PMC5051824 27706213

[106] Nguyen LT, Schmidt HA, Von Haeseler A, Minh BQ. IQ-TREE: a fast and effective stochastic algorithm for estimating maximum-likelihood phylogenies. Mol Biol Evol. 2015;32(1):268–74. 10.1093/molbev/msu300 PMC4271533 25371430

[107] Revell LJ. phytools: an R package for phylogenetic comparative biology (and other things). Methods Ecol Evol. 2012;3(2):217–23.

