# Supplementary material for: A comparative analysis of mitochondrial ORFs provides new insights on expansion of mitochondrial genome size in Arcidae
Source: BMC Genomics. 2022 Dec 7;23:809. doi: 10.1186/s12864-022-09040-3 (PMC9727918; doi:10.1186/s12864-022-09040-3)
Supplement: Supplementary file 1 — Additional file 1. Table S1-S4. Table S5. Table S6. Table S7-S12 and S14. Table S13. [file 12864_2022_9040_MOESM1_ESM.zip › Additional file 1 Table S5 .docx]

**Table S5**

BLAST results of possible duplicated ORF in *Scapharca broughtonii* (3). Since ORF protein sequences vary little within a species, only ORF protein sequences was in *Scapharca broughtonii* (3) used for BLAST and display.

| Query Name | Target Name |  | E-value | Score | Identities | Positives |
| --- | --- | --- | --- | --- | --- | --- |
| ORF8 | ORF11 |  | 1e-55 | 166 bits (421) | 90/193 (47%) | 124/193 (64%) |
|  | ORF7 |  | 2e-22 | 81.3 bits (199) | 51/131 (39%) | 79/131 (60%) |
|  | ORF78 |  | 7e-18 | 68.6 bits (166) | 61/202 (30%) | 95/202 (47%) |
| ORF127 | ORF87 |  | 0 | 835 bits (2158) | 433/648 (67%) | 501/648 (77%) |
| ORF104 | ORF46 |  | 5e-37 | 117 bits (292) | 69/160 (43%) | 93/160 (58%) |
|  | ORF106 |  | 6e-23 | 80.5 bits (197) | 50/156 (32%) | 76/156 (49%) |
|  | ORF49 |  | 7e-10 | 45.4 bits (106) | 39/137 (28%) | 65/137 (47%) |

**Table S5-b**

Annotation of possible duplicated ORFs in four *Scapharca broughtonii* mitogenomes. Sb1, *Scapharca broughtonii* (1). The rest of the abbreviations are the same.

| ORF | Length(bp) | | | |  | Initiation and termination codon | | | |
| --- | --- | --- | --- | --- | --- | --- | --- | --- | --- |
|  | Sb1 | Sb2 | Sb3 | Sb4 |  | Sb1 | Sb2 | Sb3 | Sb4 |
| ORF87 | 1983 | 1983 | 1983 | 1983 |  | ATG-TAG | ATG-TAG | ATG-TAG | ATG-TAG |
| ORF127 | 1809 | 1872 | 1872 | 1872 |  | ATT-TAG | ATG-TAG | ATG-TAG | ATG-TAG |
| ORF7 | 693 | 693 | 693 | 645 |  | ATG-TAG | ATG-TAG | ATG-TAG | ATG-TAG |
| ORF8 | 636 | 561 | 636 | 609 |  | ATA-TAA | ATG-TAA | ATA-TAA | ATA-TAA |
| ORF78 | 615 | 615 | 615 | 615 |  | ATC-TAA | ATC-TAA | ATC-TAA | ATC-TAA |
| ORF11 | 603 | 603 | 603 | 603 |  | ATA-TAG | ATA-TAG | ATA-TAG | ATA-TAG |
| ORF104 | 513 | 513 | 513 | 513 |  | ATA-TAG | ATA-TAG | ATA-TAG | ATA-TAG |
| ORF106 | 576 | 576 | 576 | 591 |  | GTG-TAA | GTG-TAA | GTG-TAA | ATG-TAA |
| ORF46 | 576 | 576 | 576 | 534 |  | ATG-TAA | ATG-TAA | ATG-TAA | ATT-TAA |
| ORF49 | 615 | 615 | 615 | 615 |  | ATT-TAG | ATT-TAG | ATT-TAG | ATC-TAA |
