# Supplementary material for: A comparative analysis of mitochondrial ORFs provides new insights on expansion of mitochondrial genome size in Arcidae
Source: BMC Genomics. 2022 Dec 7;23:809. doi: 10.1186/s12864-022-09040-3 (PMC9727918; doi:10.1186/s12864-022-09040-3)
Supplement: Supplementary file 2 — Additional file 2. R pipeline and TPM file. [file 12864_2022_9040_MOESM2_ESM.pdf]

Additional file 2: R pipeline and TPM file

R pipeline for FPKM calculation:

```
count <-read.csv("count_with_length.csv")

count <- count [,-1]

kb <- count $Length/1000

countdata <- count [,2 : 4]

rpk <- countdata/kb

tpm <- t(t(rpk)/colSums(rpk) * 1000000)

write.csv(tpm,file="tpm_genesandORFs.csv")
```

count_with_length.csv (Mapped read counts) and tpm_genesandORFs.csv (TPM value) are showed on the next page.

count_with_length.csv: the file from featureCounts results

	gene	S1	S2	S3	Length
1	Cox1	86763	157084	128635	1584
2	Cox2	149761	181509	133546	720
3	Cox3	105054	136760	90154	759
4	Nad3	70740	92613	80090	408
5	Nad4L	42299	40874	39562	276
6	Nad5	197872	345288	260600	1707
7	Nad6	115585	202194	158946	468
8	Atp6	38551	66625	28215	675
9	Cob	53102	105341	68664	1278
10	Nad1	39127	55001	34846	975
11	Nad2	27339	72259	23435	1059
12	Nad4	26957	38519	27971	1296
13	ORF87	4265	8519	6538	1983
14	ORF40	6937	11861	7145	819
15	ORF104	495	437	255	513
16	ORF8	12648	18169	4873	609
17	ORF11	7374	6036	6839	603
18	ORF10	2894	2486	1827	585
19	ORF127	1912	1624	1472	1809
20	ORF5	5270	10275	8399	762
21	ORF86	6014	3166	3494	582
22	ORF21	3493	1437	3223	239
23	ORF103	489	355	239	246

tpm_genesandORFs.csv: the TPM file from R pipeline result

	S1	S2	S3
Cox1	40218.96116	50376.80365	55576.6823
Cox2	152727.661	128061.7705	126936.6498
Cox3	101630.0836	91531.62031	81289.0164
Nad3	127308.1943	115309.6419	134340.4425
Nad4L	112531.0792	75230.14393	98097.4686
Nad5	85114.25226	102754.7243	104479.1268
Nad6	181345.6281	219470.5465	232430.1133
Atp6	41935.64633	50140.34102	28606.51321
Cob	30509.2471	41871.68298	36769.42934
Nad1	29466.15035	28656.27948	24458.90221
Nad2	18955.62597	34661.69697	15144.59423
Nad4	15272.77743	15098.1535	14770.37888
ORF87	1579.238733	2182.32656	2256.37275
ORF40	6219.267811	7356.83877	5970.448759
ORF104	708.4990947	432.7308981	340.1821012
ORF8	15249.51495	15155.40569	5476.054513
ORF11	8979.192597	5084.939067	7761.826587
ORF10	3632.403843	2158.733805	2137.328738
ORF127	776.0698059	456.0382729	556.8752126
ORF5	5078.168529	6849.844197	7543.297809
ORF86	7587.381214	2763.387317	4108.549843
ORF21	10731.29542	3054.307097	9228.923554
ORF103	1459.570973	733.0727568	664.8925057
