# Supplementary material for: A comparative analysis of mitochondrial ORFs provides new insights on expansion of mitochondrial genome size in Arcidae
Source: BMC Genomics. 2022 Dec 7;23:809. doi: 10.1186/s12864-022-09040-3 (PMC9727918; doi:10.1186/s12864-022-09040-3)

Additional file 4: ORF Sequence alignment

ORF5:

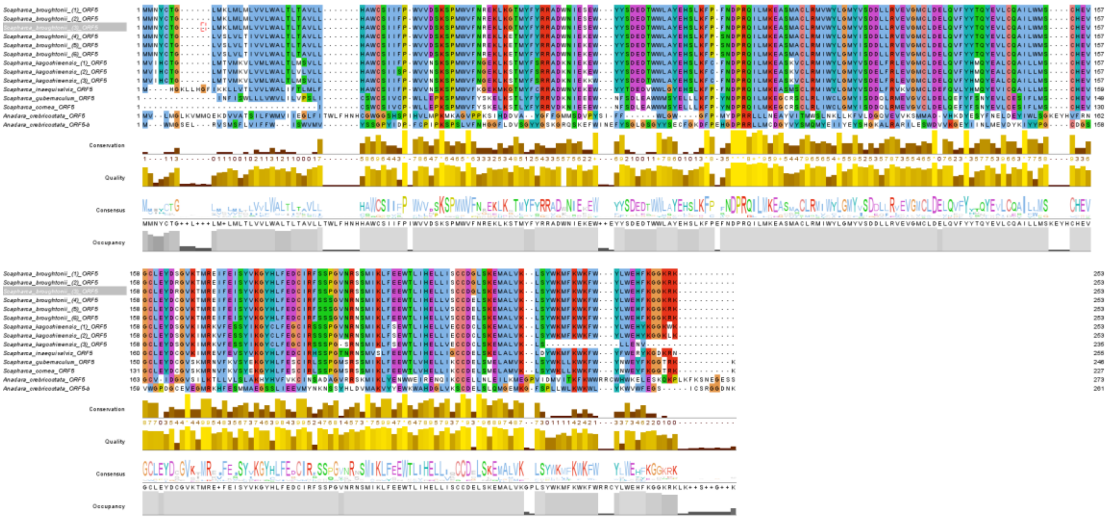

ORF7:

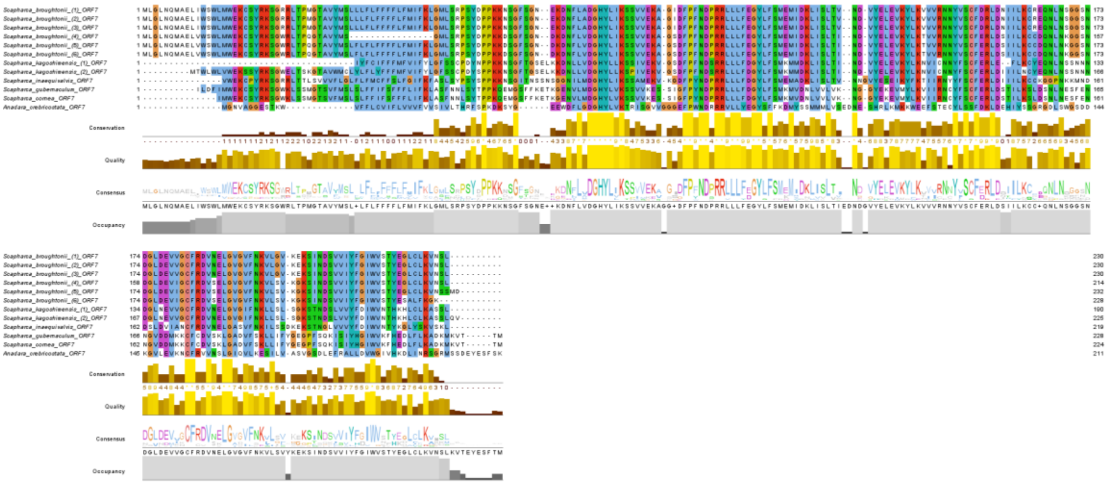

ORF8:

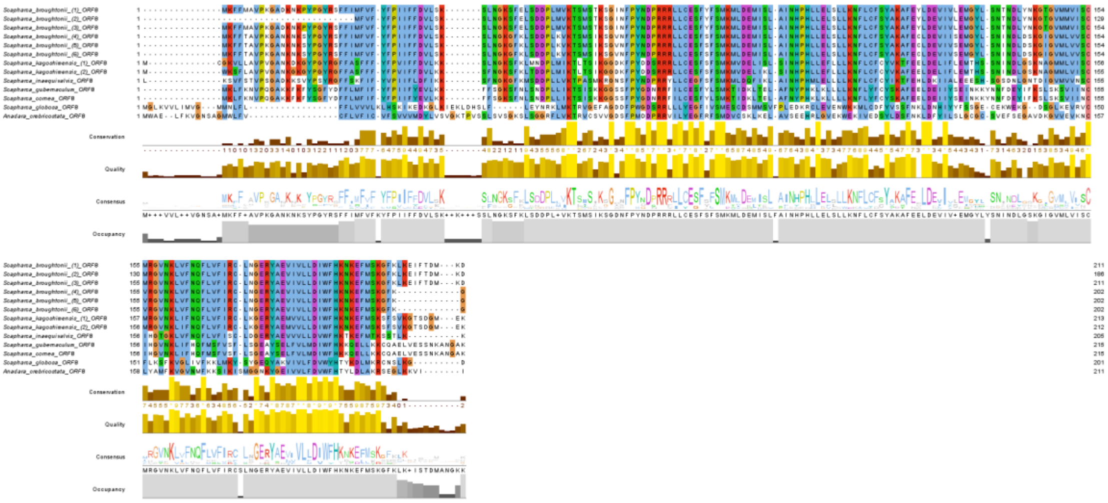

ORF10:

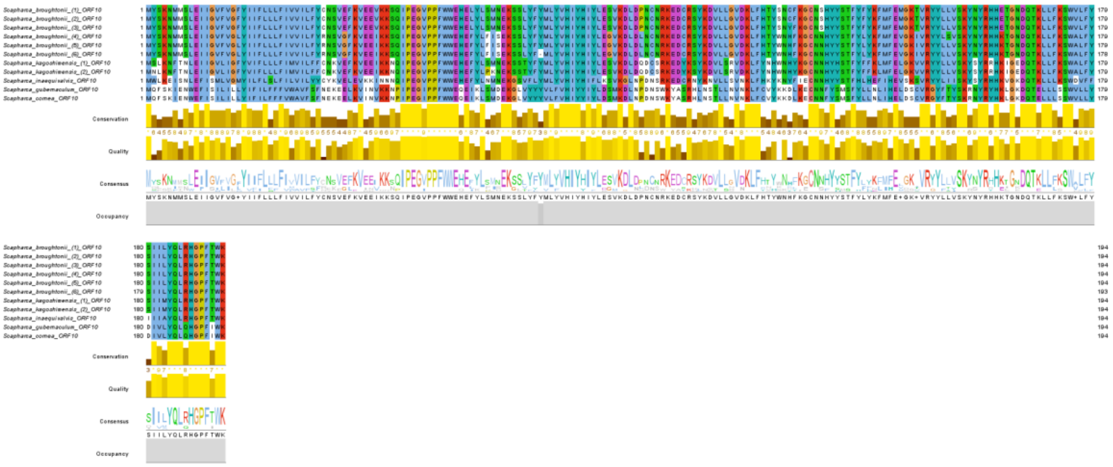

ORF11:

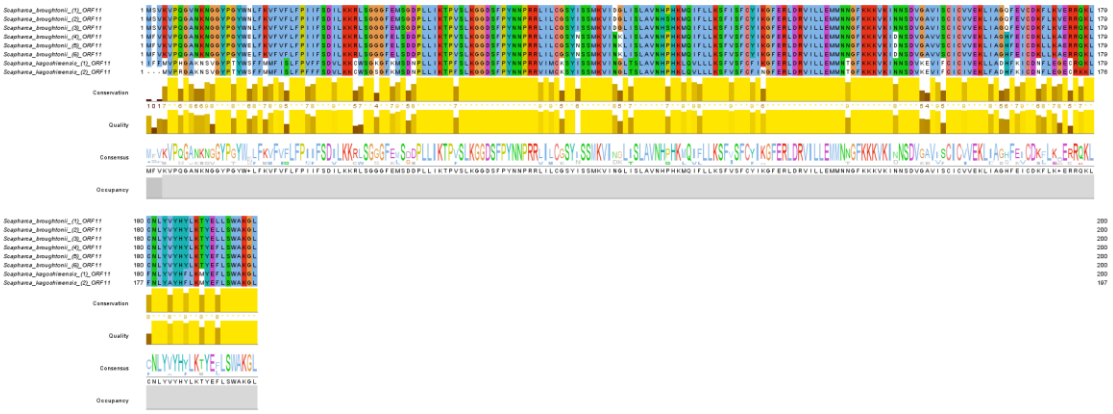

ORF40:

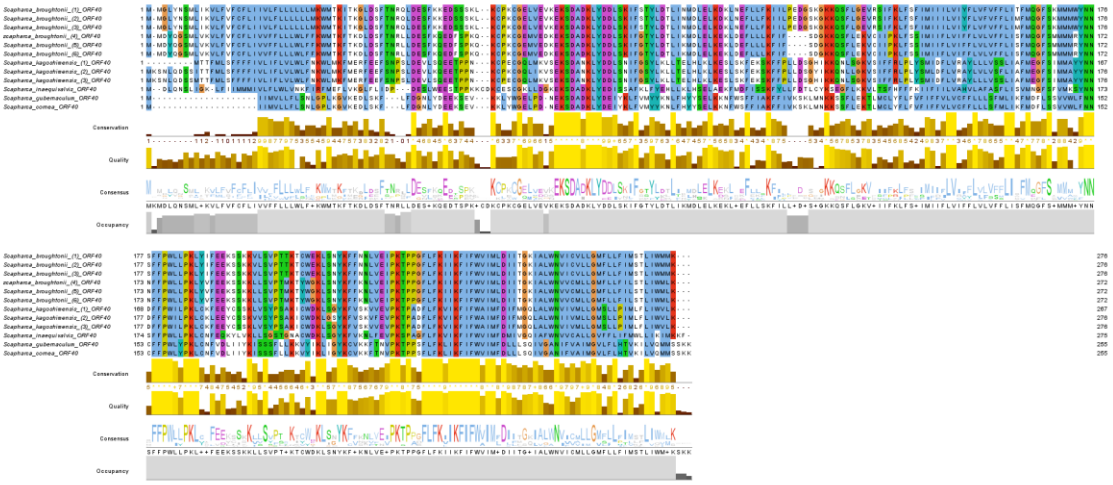

ORF46:

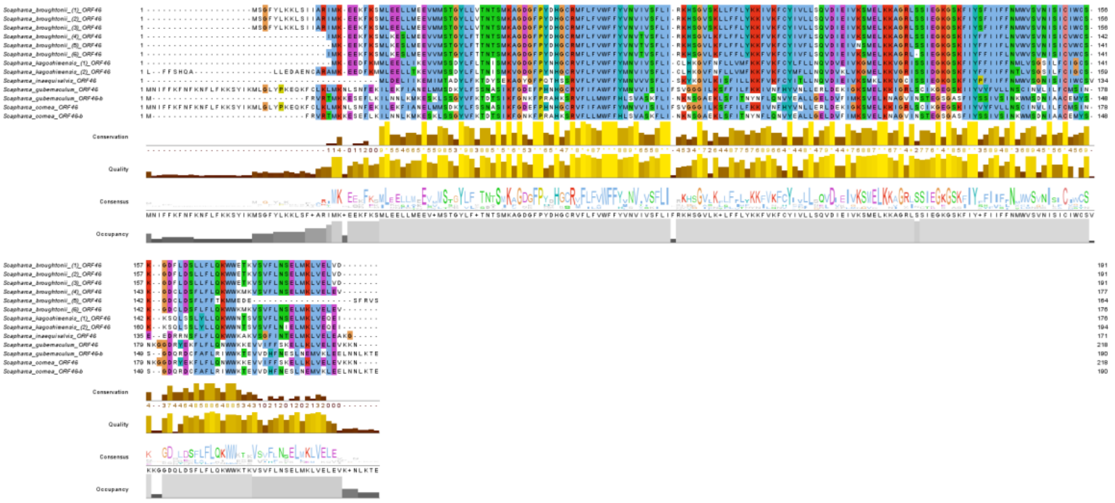

ORF49:

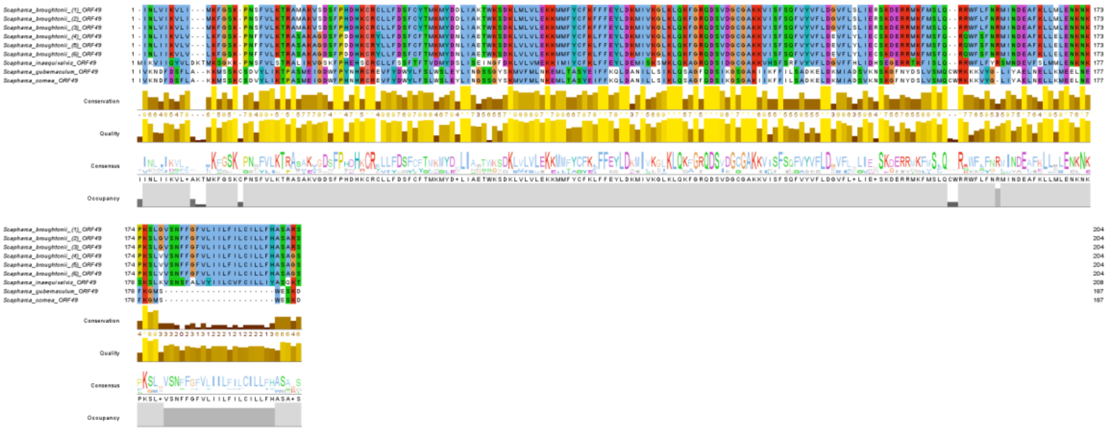

ORF78:

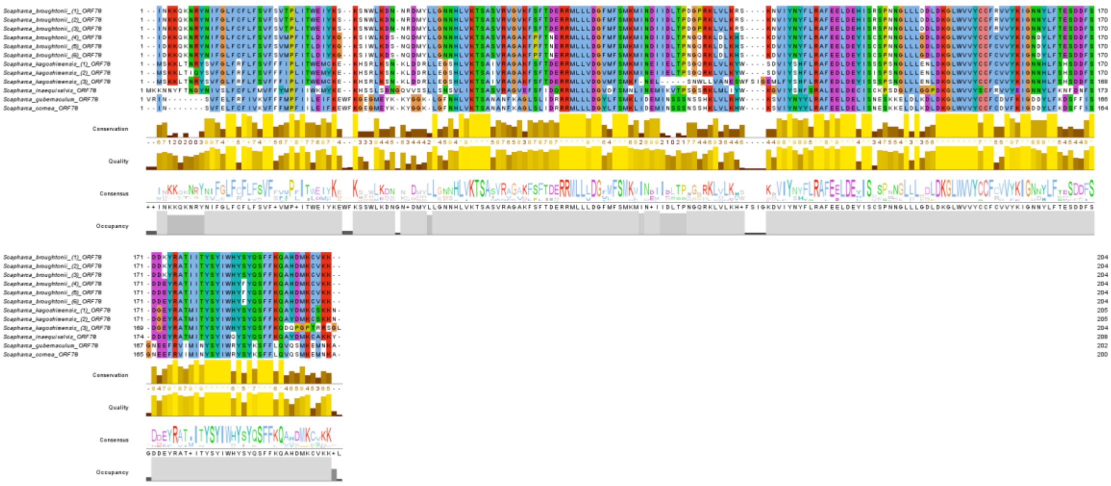

ORF86:

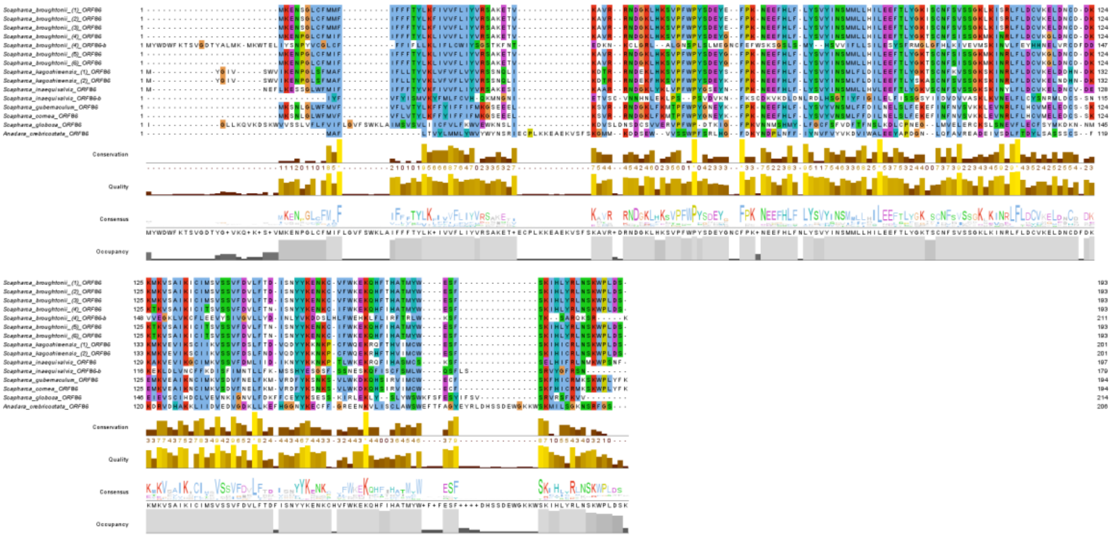

ORF87:

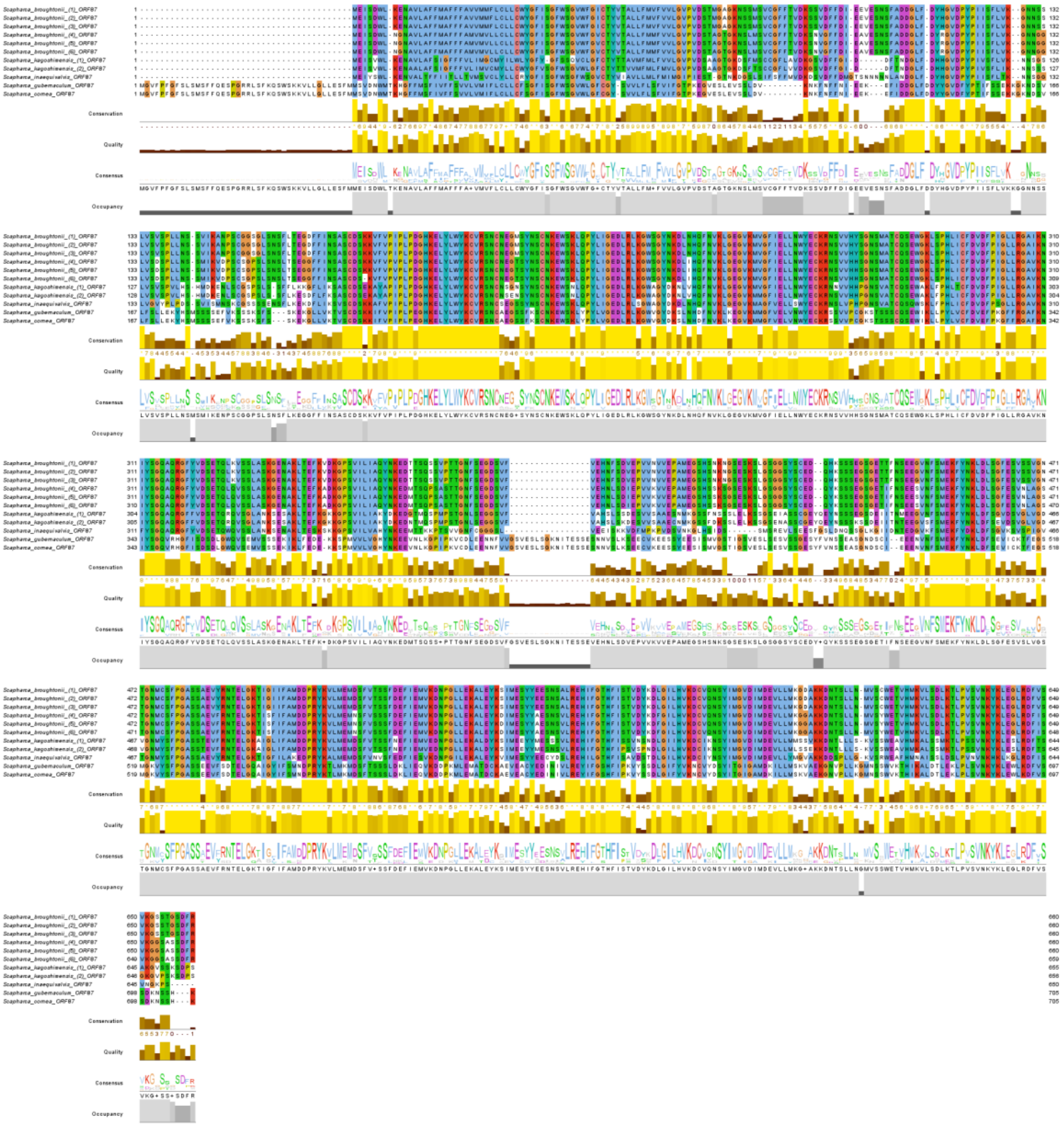

ORF104:

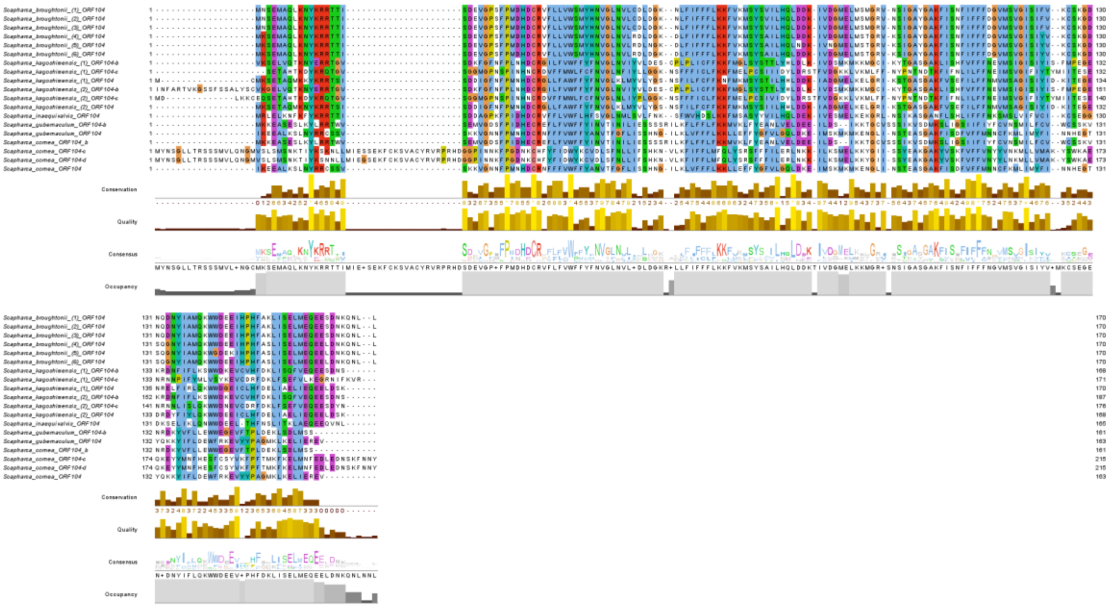

ORF106:

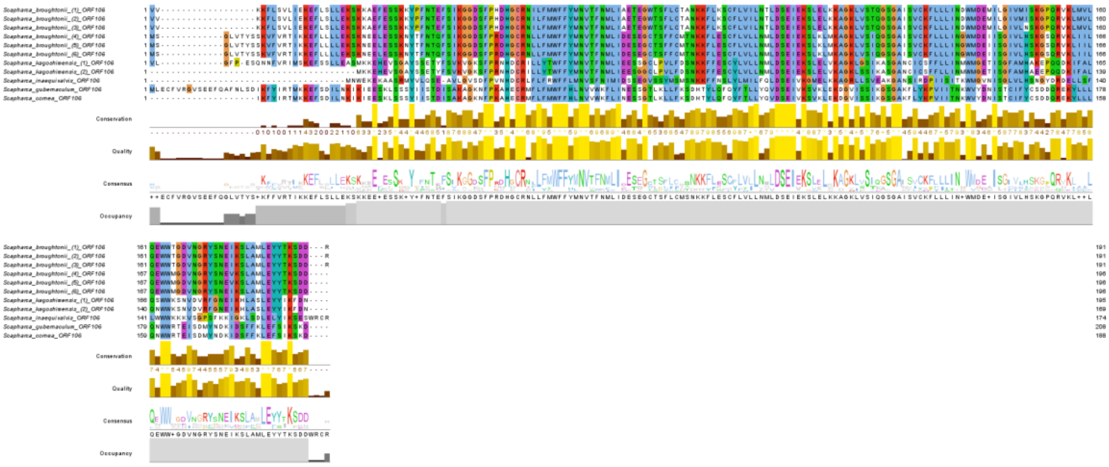

ORF127:

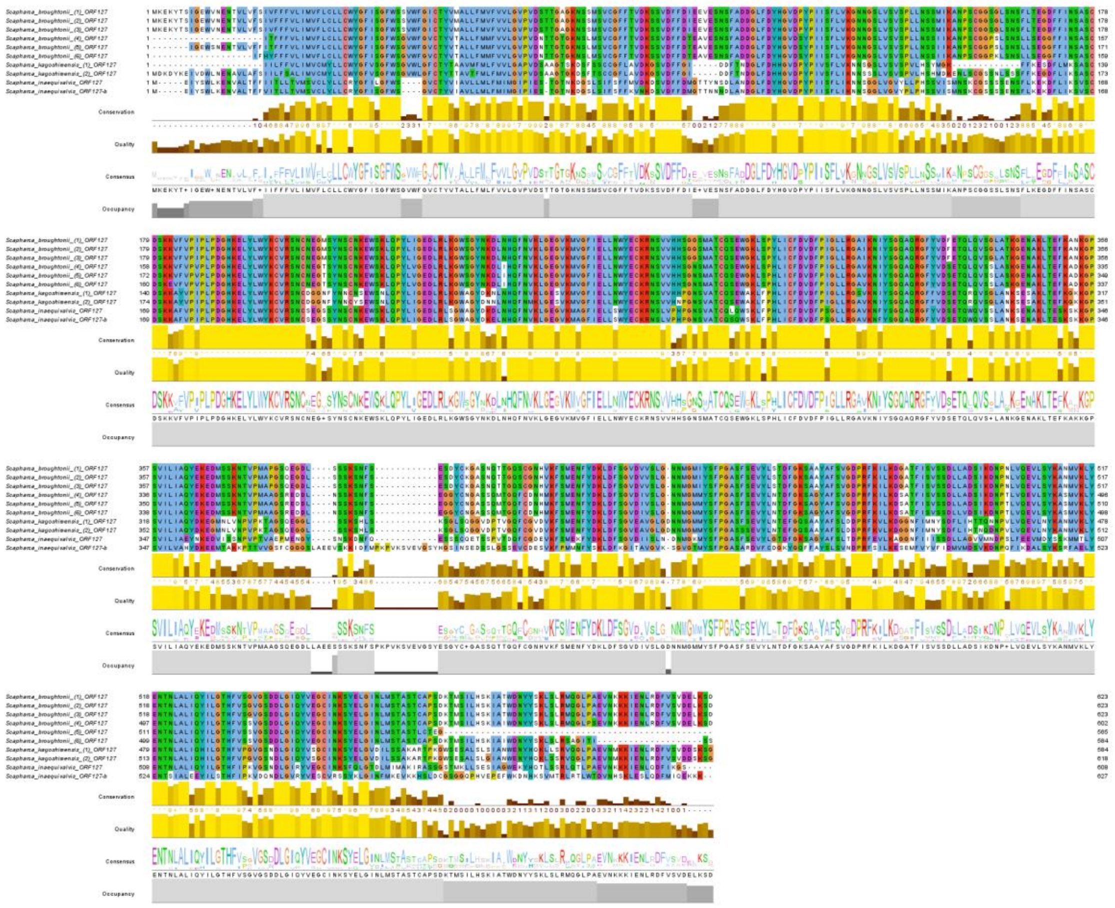

ORF21:

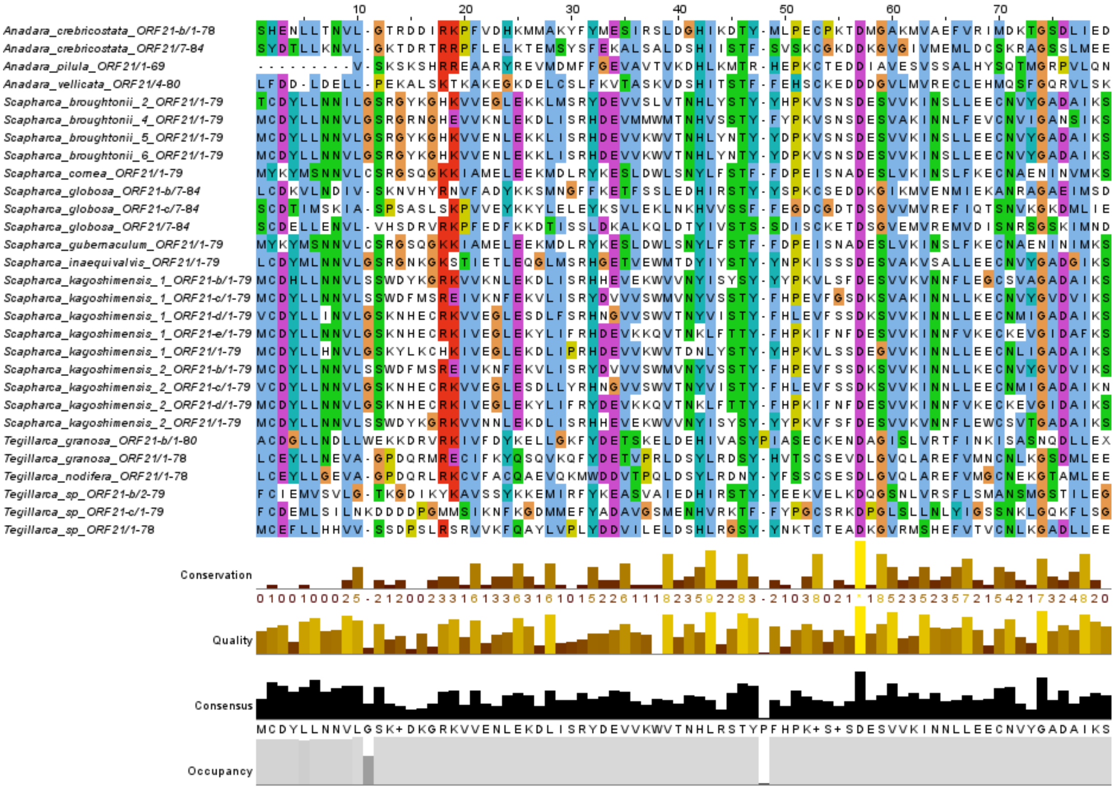

ORF103:

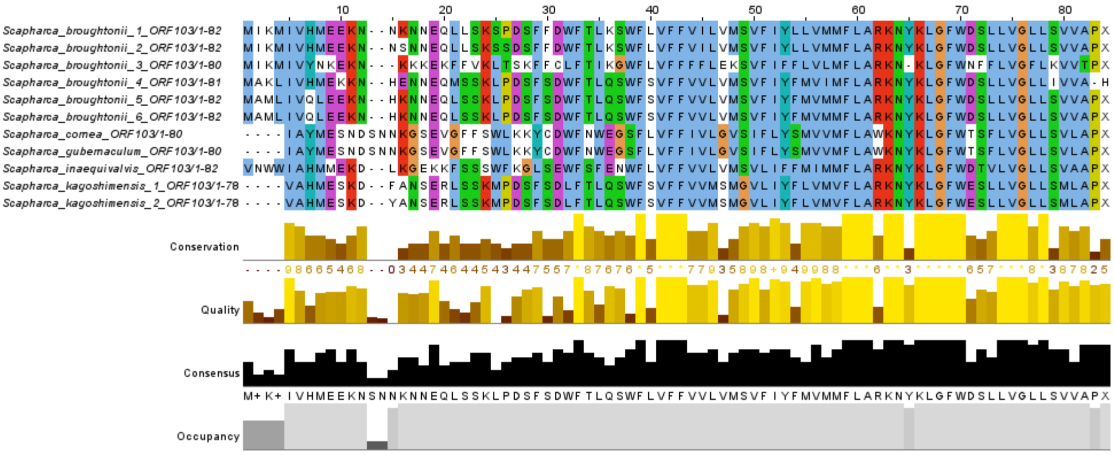

Supplement: Supplementary file 4 — Additional file 4. ORF sequence alignment. [file 12864_2022_9040_MOESM4_ESM.pdf]
