# Supplementary material for: A comparative analysis of mitochondrial ORFs provides new insights on expansion of mitochondrial genome size in Arcidae
Source: BMC Genomics. 2022 Dec 7;23:809. doi: 10.1186/s12864-022-09040-3 (PMC9727918; doi:10.1186/s12864-022-09040-3)
Supplement: Supplementary file 5 — Additional file 5 The Blastn and Blastx results of Scapharca broughtonii ORFs with nuclear genome. [file 12864_2022_9040_MOESM5_ESM.zip › Additional file 5-a Scapharca_broughtonii_ORF_nucleargenome_blastn.pdf]

Additional file 5-a: the blastn result of Scapharca broughtonii ORFs with its nuclear genome

BLASTN 2.7.1+

Reference: Zheng Zhang, Scott Schwartz, Lukas Wagner, and Webb
Miller (2000), "A greedy algorithm for aligning DNA sequences", J
Comput Biol 2000; 7(1-2):203-14.

Database: ../Scapharca_broughtonii.genomic.fa
1,026 sequences; 884,566,040 total letters

Query= Scapharca_broughtonii_(4)_ORF104

Length=513

***** No hits found *****

Lambda K H
1.33 0.621 1.12

Gapped
Lambda K H
1.28 0.460 0.850

Effective search space used: 429000596320

Query= Scapharca_broughtonii_(4)_ORF106

Length=591

***** No hits found *****

Lambda K H
1.33 0.621 1.12

Gapped
Lambda K H
1.28 0.460 0.850

Effective search space used: 497994506656

Query= Scapharca_broughtonii_(4)_ORF10

Length=585

***** No hits found *****

Lambda K H
1.33 0.621 1.12

Gapped
Lambda K H
1.28 0.460 0.850

Effective search space used: 492687282784

Query= Scapharca_broughtonii_(4)_ORF11

Length=603

***** No hits found *****

Lambda K H
1.33 0.621 1.12

Gapped
Lambda K H
1.28 0.460 0.850

Effective search space used: 508608954400

Query= Scapharca_broughtonii_(4)_ORF127

Length=1809

***** No hits found *****

Lambda	K	H
1.33	0.621	1.12

Gapped

Lambda	K	H
1.28	0.460	0.850

Effective search space used: 1573588227540

Query= Scapharca_broughtonii_(4)_ORF40

Length=819

***** No hits found *****

Lambda	K	H
1.33	0.621	1.12

Gapped

Lambda	K	H
1.28	0.460	0.850

Effective search space used: 698783665940

Query= Scapharca_broughtonii_(4)_ORF46

Length=534

***** No hits found *****

Lambda K H
1.33 0.621 1.12

Gapped
Lambda K H
1.28 0.460 0.850

Effective search space used: 447575879872

Query= Scapharca_broughtonii_(4)_ORF49

Length=615

***** No hits found *****

Lambda K H
1.33 0.621 1.12

Gapped
Lambda K H
1.28 0.460 0.850

Effective search space used: 519223402144

Query= Scapharca_broughtonii_(4)_ORF5

Length=762

***** No hits found *****

Lambda K H

1.33	0.621	1.12

Gapped

Lambda	K	H
1.28	0.460	0.850

Effective search space used: 649250387008

Query= Scapharca_broughtonii_(4)_ORF78

Length=615

***** No hits found *****

Lambda	K	H
1.33	0.621	1.12

Gapped

Lambda	K	H
1.28	0.460	0.850

Effective search space used: 519223402144

Query= Scapharca_broughtonii_(4)_ORF7

Length=645

***** No hits found *****

Lambda	K	H
1.33	0.621	1.12

Gapped

Lambda	K	H
1.28	0.460	0.850

Effective search space used: 545759521504

Query= Scapharca_broughtonii_(4)_ORF86

Length=582

***** No hits found *****

Lambda K H
1.33 0.621 1.12

Gapped
Lambda K H
1.28 0.460 0.850

Effective search space used: 490033670848

Query= Scapharca_broughtonii_(4)_ORF87

Length=1983

***** No hits found *****

Lambda K H
1.33 0.621 1.12

Gapped
Lambda K H
1.28 0.460 0.850

Effective search space used: 1727497362780

Query= Scapharca_broughtonii_(4)_ORF8

Length=609

***** No hits found *****

Lambda K H
1.33 0.621 1.12

Gapped
Lambda K H
1.28 0.460 0.850

Effective search space used: 513916178272

Database: ../Scapharca_broughtonii.genomic.fa
Posted date: Aug 29, 2021 10:16 AM
Number of letters in database: 884,566,040
Number of sequences in database: 1,026

Matrix: blastn matrix 1 -2
Gap Penalties: Existence: 0, Extension: 2.5
