# Supplementary material for: A comparative analysis of mitochondrial ORFs provides new insights on expansion of mitochondrial genome size in Arcidae
Source: BMC Genomics. 2022 Dec 7;23:809. doi: 10.1186/s12864-022-09040-3 (PMC9727918; doi:10.1186/s12864-022-09040-3)
Supplement: Supplementary file 5 — Additional file 5 The Blastn and Blastx results of Scapharca broughtonii ORFs with nuclear genome. [file 12864_2022_9040_MOESM5_ESM.zip › Additional file 5-b Scapharca_broughtonii_ORF_nucleargenome_blastx.pdf]

Additional file 5-b: the blastx result of Scapharca broughtonii ORFs with its nuclear genome

BLASTX 2.7.1+

Reference: Stephen F. Altschul, Thomas L. Madden, Alejandro A. Schaffer, Jinghui Zhang, Zheng Zhang, Webb Miller, and David J. Lipman (1997), "Gapped BLAST and PSI-BLAST: a new generation of protein database search programs", Nucleic Acids Res. 25:3389-3402.

Database: ../Scapharca_broughtonii.pep.fa
24,045 sequences; 12,762,346 total letters

Query= Scapharca_broughtonii_(4)_ORF104

Length=111

***** No hits found *****

Lambda	K	H	a	alpha
0.318	0.134	0.401	0.792	4.96

Gapped

Lambda	K	H	a	alpha	sigma
0.267	0.0410	0.140	1.90	42.6	43.6

Effective search space used: 312446275

Query= Scapharca_broughtonii_(4)_ORF106

Length=126

***** No hits found *****

Lambda K H a alpha

0.300	0.0852	0.175	0.792	4.96

Gapped

Lambda	K	H	a	alpha	sigma
0.267	0.0410	0.140	1.90	42.6	43.6

Effective search space used: 321818276

Query= Scapharca_broughtonii_(4)_ORF10

Length=124

***** No hits found *****

Lambda	K	H	a	alpha
0.318	0.134	0.401	0.792	4.96

Gapped

Lambda	K	H	a	alpha	sigma
0.267	0.0410	0.140	1.90	42.6	43.6

Effective search space used: 322443446

Query= Scapharca_broughtonii_(4)_ORF11

Length=127

***** No hits found *****

Lambda	K	H	a	alpha
0.305	0.169	0.616	0.792	4.96

Gapped

Lambda	K	H	a	alpha	sigma
0.267	0.0410	0.140	1.90	42.6	43.6

Effective search space used: 321818276

Query= Scapharca_broughtonii_(4)_ORF127

Length=420

***** No hits found *****

Lambda	K	H	a	alpha
0.318	0.134	0.401	0.792	4.96

Gapped

Lambda	K	H	a	alpha	sigma
0.267	0.0410	0.140	1.90	42.6	43.6

Effective search space used: 529914800

Query= Scapharca_broughtonii_(4)_ORF40

Length=147

***** No hits found *****

Lambda	K	H	a	alpha
0.318	0.134	0.401	0.792	4.96

Gapped

Lambda	K	H	a	alpha	sigma
0.267	0.0410	0.140	1.90	42.6	43.6

Effective search space used: 317442086

Query= Scapharca_broughtonii_(4)_ORF46

Length=112

***** No hits found *****

Lambda	K	H	a	alpha
0.300	0.0852	0.175	0.792	4.96

Gapped

Lambda	K	H	a	alpha	sigma
0.267	0.0410	0.140	1.90	42.6	43.6

Effective search space used: 311845150

Query= Scapharca_broughtonii_(4)_ORF49

Length=123

***** No hits found *****

Lambda	K	H	a	alpha
0.303	0.107	0.219	0.792	4.96

Gapped

Lambda	K	H	a	alpha	sigma
0.267	0.0410	0.140	1.90	42.6	43.6

Effective search space used: 322443446

Query= Scapharca_broughtonii_(4)_ORF5

Length=169

***** No hits found *****

Lambda	K	H	a	alpha
0.318	0.134	0.401	0.792	4.96

Gapped

Lambda	K	H	a	alpha	sigma
0.267	0.0410	0.140	1.90	42.6	43.6

Effective search space used: 313065896

Query= Scapharca_broughtonii_(4)_ORF78

Length=136

***** No hits found *****

Lambda	K	H	a	alpha
0.285	0.112	0.343	0.792	4.96

Gapped

Lambda	K	H	a	alpha	sigma
0.267	0.0410	0.140	1.90	42.6	43.6

Effective search space used: 319942766

Query= Scapharca_broughtonii_(4)_ORF7

Length=149

***** No hits found *****

Lambda	K	H	a	alpha
0.318	0.134	0.401	0.792	4.96

Gapped

Lambda	K	H	a	alpha	sigma
0.267	0.0410	0.140	1.90	42.6	43.6

Effective search space used: 317442086

Query= Scapharca_broughtonii_(4)_ORF86

Length=126

***** No hits found *****

Lambda	K	H	a	alpha
0.300	0.0852	0.175	0.792	4.96

Gapped

Lambda	K	H	a	alpha	sigma
0.267	0.0410	0.140	1.90	42.6	43.6

Effective search space used: 321818276

Query= Scapharca_broughtonii_(4)_ORF87

Length=459

***** No hits found *****

Lambda	K	H	a	alpha
0.318	0.134	0.401	0.792	4.96

Gapped

Lambda	K	H	a	alpha	sigma
0.267	0.0410	0.140	1.90	42.6	43.6

Effective search space used: 655603562

Query= Scapharca_broughtonii_(4)_ORF8

Length=127

***** No hits found *****

Lambda	K	H	a	alpha
0.297	0.129	0.386	0.792	4.96

Gapped

Lambda	K	H	a	alpha	sigma
0.267	0.0410	0.140	1.90	42.6	43.6

Effective search space used: 322443446

Database: ../Scapharca_broughtonii.pep.fa
Posted date: Oct 20, 2021 9:49 PM
Number of letters in database: 12,762,346
Number of sequences in database: 24,045

Matrix: BLOSUM62
Gap Penalties: Existence: 11, Extension: 1
Neighboring words threshold: 12
Window for multiple hits: 40
