# Supplementary material for: A comparative analysis of mitochondrial ORFs provides new insights on expansion of mitochondrial genome size in Arcidae
Source: BMC Genomics. 2022 Dec 7;23:809. doi: 10.1186/s12864-022-09040-3 (PMC9727918; doi:10.1186/s12864-022-09040-3)

Additional file 6: the tree of ORF7, ORF8, ORF78 and ORF11

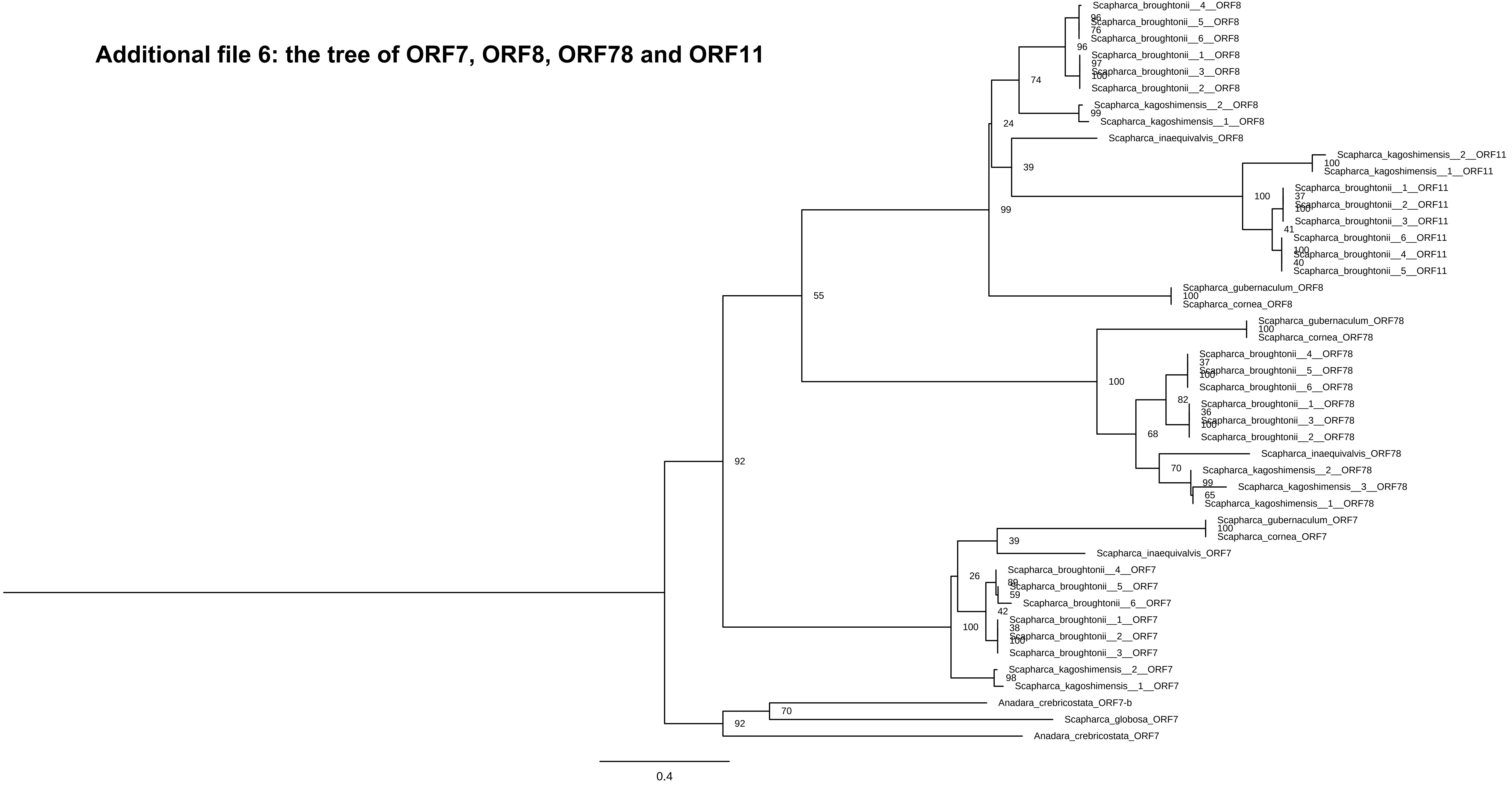

Additional file 4: the tree of ORF104, ORF106, ORF46 and ORF49

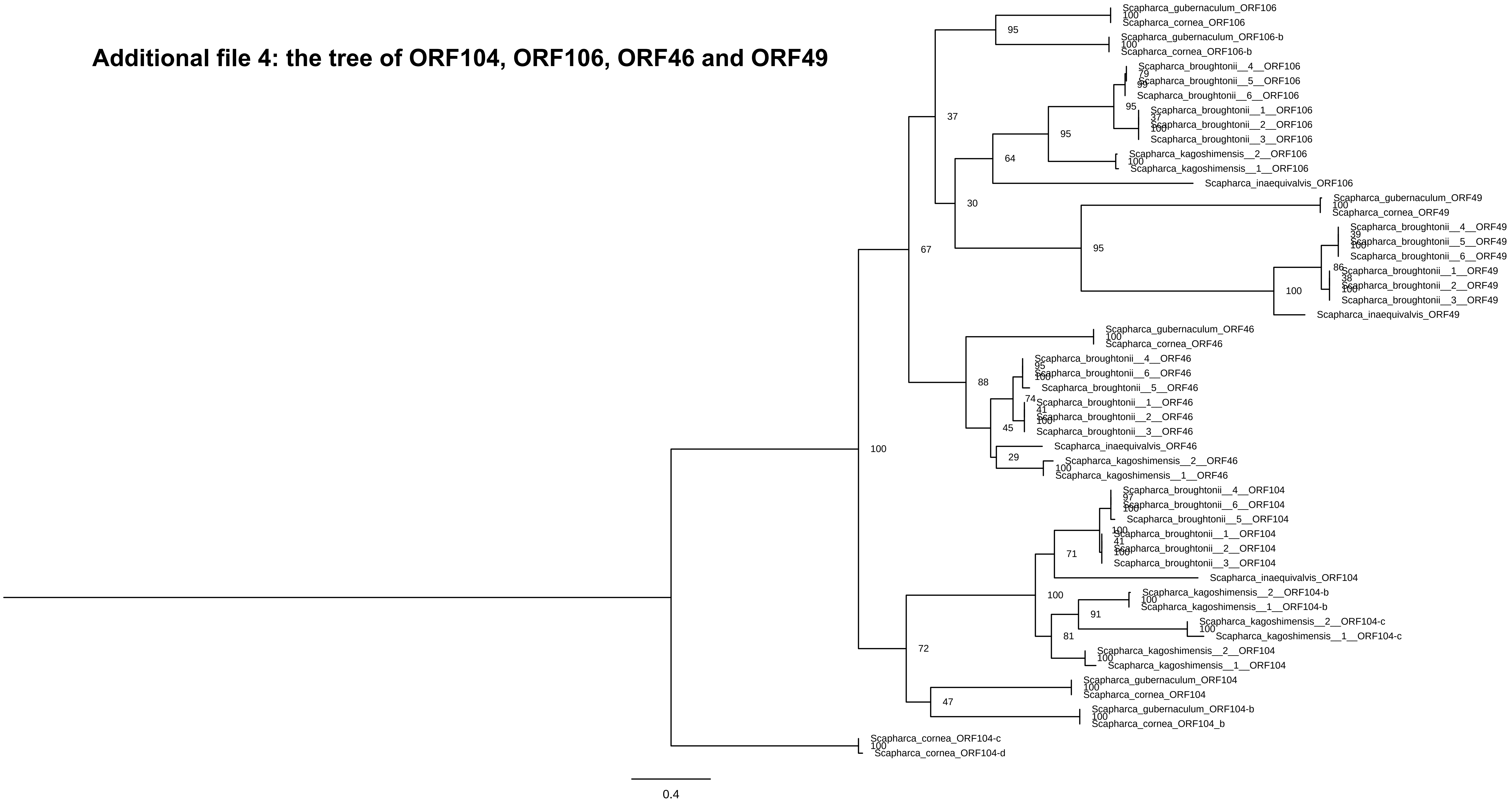

Additional file 4: the tree of ORF87 and ORF127

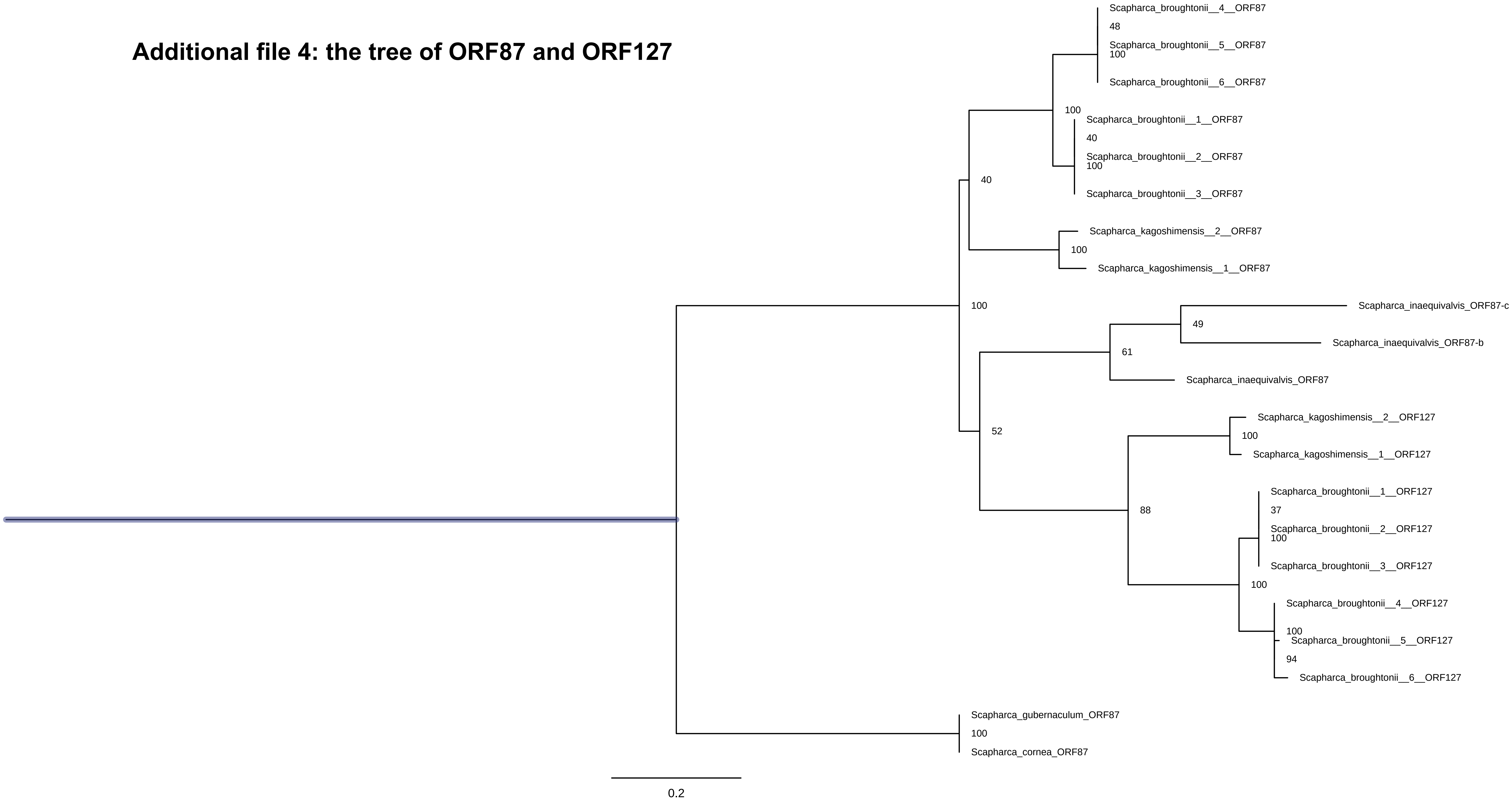

Supplement: Supplementary file 6 — Additional file 6. The tree of duplicated ORFs. [file 12864_2022_9040_MOESM6_ESM.pdf]
