# Supplementary material for: A comparative analysis of mitochondrial ORFs provides new insights on expansion of mitochondrial genome size in Arcidae
Source: BMC Genomics. 2022 Dec 7;23:809. doi: 10.1186/s12864-022-09040-3 (PMC9727918; doi:10.1186/s12864-022-09040-3)

Additional file 7： Secondary structure and relative solvent accessibility of ATP8 and ORFs.

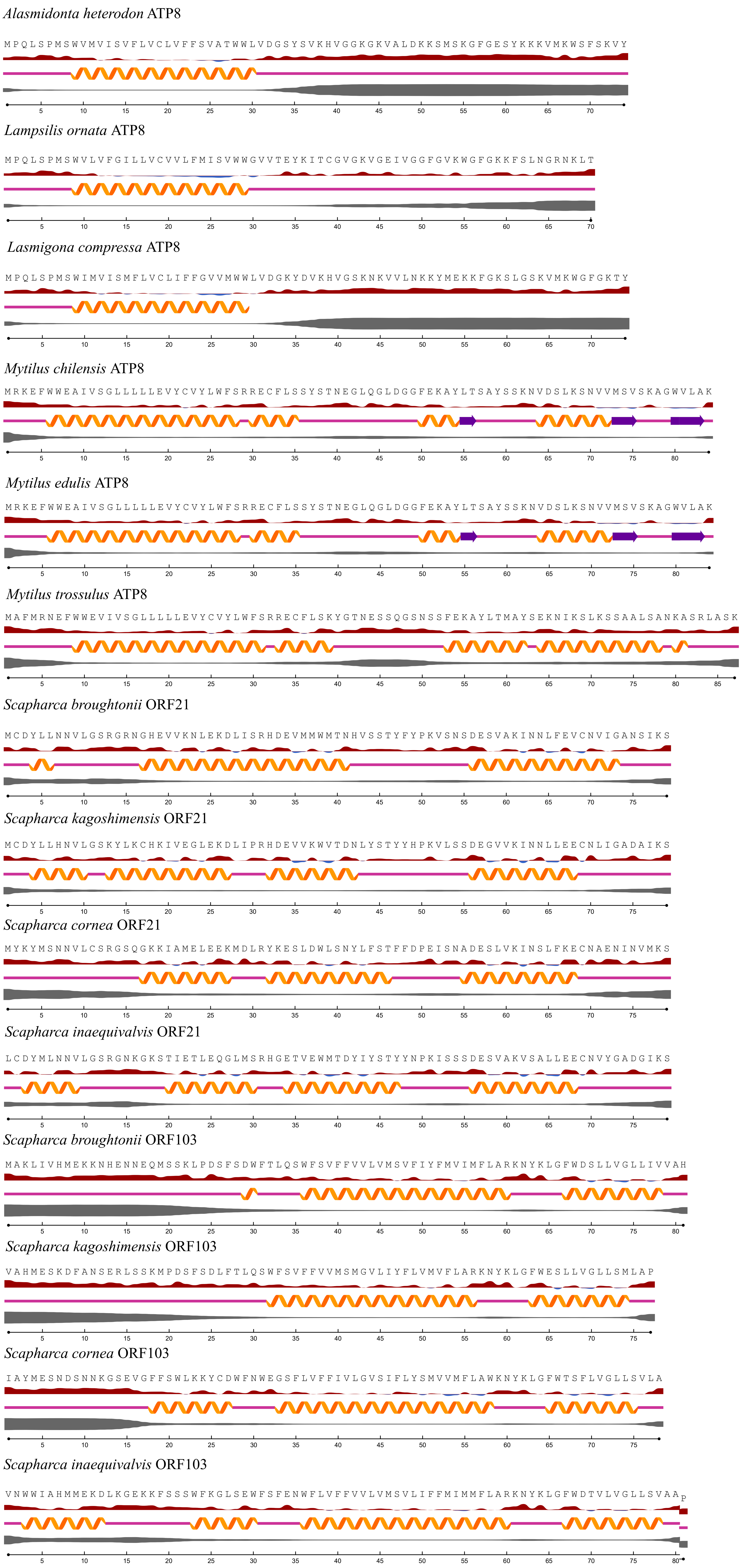

Supplement: Supplementary file 7 — Additional file 7. Secondary structure and relative solvent accessibility of ATP8 and ORFs. [file 12864_2022_9040_MOESM7_ESM.pdf]
